# Low expression of T-cell transcription factor *BCL11b* predicts inferior survival in adult standard risk T-cell acute lymphoblastic leukemia patients

**DOI:** 10.1186/s13045-014-0051-y

**Published:** 2014-07-15

**Authors:** Isabelle Bartram, Nicola Gökbuget, Cornelia Schlee, Sandra Heesch, Lars Fransecky, Stefan Schwartz, Reingard Stuhlmann, Kerstin Schäfer-Eckhart, Michael Starck, Albrecht Reichle, Dieter Hoelzer, Claudia D Baldus, Martin Neumann

**Affiliations:** 1 Department of Hematology and Oncology, Charité, University Hospital Berlin, Campus Benjamin Franklin, Hindenburgdamm 30, Berlin, 12203, Germany; 2 Department of Medicine II, Hematology/Oncology, Goethe University Hospital, Frankfurt/M, Germany; 3 Department of Hematology, Oncology and Stem cell transplantation, Asklepios Klinik St. Georg, Asklepios Klinik St. Georg, Hamburg, Germany; 4 Department of Hematology and Oncology, Klinikum Nürnberg, Nürnberg, Germany; 5 Department of Hematology and Oncology, Klinikum Schwabing, München, Germany; 6 Department for Internal Medicine III, University Hospital Regensburg, Regensburg, Germany

**Keywords:** Adult T-cell acute lymphoblastic leukemia, BCL11b, Outcome, Standard risk, Expression, Mutation

## Abstract

**Background:**

Risk stratification, detection of minimal residual disease (MRD), and implementation of novel therapeutic agents have improved outcome in acute lymphoblastic leukemia (ALL), but survival of adult patients with T-cell acute lymphoblastic leukemia (T-ALL) remains unsatisfactory. Thus, novel molecular insights and therapeutic approaches are urgently needed.

**Methods:**

We studied the impact of *B-cell CLL/lymphoma 11b (BCL11b)*, a key regulator in normal T-cell development, in T-ALL patients enrolled into the German Multicenter Acute Lymphoblastic Leukemia Study Group trials (GMALL; n = 169). The mutational status (exon 4) of *BCL11b* was analyzed by Sanger sequencing and mRNA expression levels were determined by quantitative real-time PCR. In addition gene expression profiles generated on the Human Genome U133 Plus 2.0 Array (affymetrix) were used to investigate *BCL11b* low and high expressing T-ALL patients.

**Results:**

We demonstrate that *BCL11b* is aberrantly expressed in T-ALL and gene expression profiles reveal an association of low *BCL11b* expression with up-regulation of immature markers. T-ALL patients characterized by low *BCL11b* expression exhibit an adverse prognosis [5-year overall survival (OS): low 35% (n = 40) vs. high 53% (n = 129), P = 0.02]. Within the standard risk group of thymic T-ALL (n = 102), low *BCL11b* expression identified patients with an unexpected poor outcome compared to those with high expression (5-year OS: 20%, n = 18 versus 62%, n = 84, P < 0.01). In addition, sequencing of exon 4 revealed a high mutation rate (14%) of *BCL11b*.

**Conclusions:**

In summary, our data of a large adult T-ALL patient cohort show that low *BCL11b* expression was associated with poor prognosis; particularly in the standard risk group of thymic T-ALL. These findings can be utilized for improved risk prediction in a significant proportion of adult T-ALL patients, which carry a high risk of standard therapy failure despite a favorable immunophenotype.

## Background

Improved treatment strategies, integrating risk stratification and minimal residual disease (MRD) monitoring, have improved survival of adult patients with acute lymphoblastic leukemia (ALL) over the last decades [[1],[2]]. Nevertheless, overall survival (OS) remains unsatisfactory with about 40-70%, depending on protocol and age group. Thus far, novel therapy approaches have mainly been developed in B-lineage ALL, where new targeted therapies with monoclonal antibodies like Rituximab or small molecule tyrosine kinase inhibitors (TKI) such as imatinib for Philadelphia chromosome/BCR-ABL-positive patients have been established [[3]–[6]]. In T-cell acute lymphoblastic leukemia (T-ALL), less progress has been made despite the introduction of nelarabine in relapsed and refractory disease [[7],[8]]. Other molecular driven approaches, including γ-secretase inhibitors, have until now been less successful [[9]].

In the German Multicenter Study Group for Adult ALL (GMALL), immunologic subtypes are routinely used as prognostic factors for the risk stratification of T-ALL. Within the high risk group of early T-ALL patients the recently identified subgroup of Early T-cell precursor (ETP-) ALL has been characterized by an immature immunophenotype with a high rate of *FLT3*-mutations, suggesting a potential role for TKI in the treatment for these high risk patients [[10]–[12]]. In contrast, T-ALL patients with a thymic phenotype have a more favorable prognosis [[1]] and are therefore classified as standard risk. Nevertheless, this large group (56% of adult T-ALL) [[3]] of standard risk T-ALL contains a molecularly and clinically heterogeneous group of patients. Thus, novel insights into the molecular stratification will further aid in refining treatment options.

**Figure 1:**
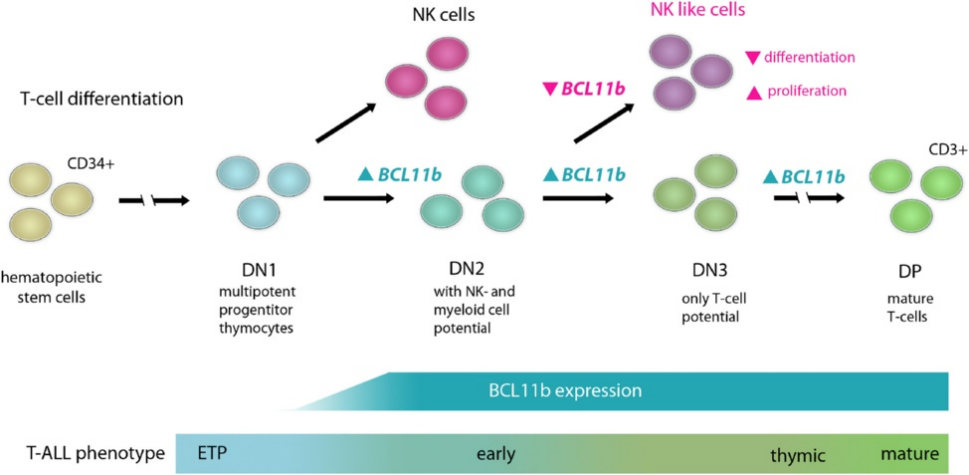
**BCL11b expression in the stages of normal T-cell development.** In the murine model BCL11b expression begins during the transition of DN1 to DN2 and continues throughout further differentiation steps and in mature T-cells [[39]]. Knockout at DN2 stage leads to a NK like phenotype with differentiation arrest and a high proliferative potential [[13]]. The corresponding human T-ALL immunophenotypes are indicated. DN = Double Negative, DP = Double Positive, NK = Natural Killer cells, ETP = Early T-cell progenitors, ▲up-regulation, ▼down-regulation.

The *B-cell CLL/lymphoma 11b* (*BCL11b*) gene, a Krüppel-like C_2_H_2_ zinkfinger transcription factor located on chromosome 14q32.2, is a key player in physiologic T-cell development with potential impact on T-ALL leukemogenesis. In normal hematopoiesis, the onset of *BCL11b* expression in T-cell progenitors occurs during the onset of DN2 phase and is maintained throughout subsequent differential stages (Figure 1) [[13],[14]]. For several target genes BCL11b acts as repressor (p21, p57), for others as activator of transcription (IL-2) [[15]–[17]]. *In vitro* studies demonstrated that knockdown inhibited proliferation and led to apoptosis in human T-ALL cell lines [[18],[19]] and a chemo-protective effect of *BCL11b* overexpression was also observed [[20]].

In addition, *BCL11b* is proposed to act as haploinsufficient tumor-suppressor, as its disruption was found to be associated with lymphomagenesis in mice [[21],[22]]. In humans, chromosomal translocations involving the *BCL11b* gene locus were identified in patients with acute myeloid leukemia (AML), T-ALL and T/myeloid acute bilineage leukemia [[23]–[28]]. Likewise, deletions and missense mutations, disrupting gene function, were reported in 9 to 16% of pediatric T-ALL patients [[29],[30]]. One study found *BCL11b* more frequently mutated in adult patients compared to children (20% vs. 5.3%) [[31]], with a lower mutation rate in early immature (3.6%) and a higher rate (12%) in cortical/mature adult T-ALL [[32]]. Studies on the prognostic impact of *BCL11b* mutations gave diverging results: a small study reported a favorable outcome for adult T-ALL patients with *BCL11b* mutations (n = 4) [[32]], however, studies in pediatric patients reported no prognostic effect of mutations [[29],[30]].

We hypothesized that deregulated expression of *BCL11b*, which is tightly regulated in normal T-cell differentiation, and *BCL11b* mutations play an important role in T-ALL. Therefore, we analyzed *BCL11b* mRNA expression levels in a large cohort of adult T-ALL patients and screened for mutations in the zinc finger region.

## Results

### *BCL11b* is heterogeneously expressed in adult T-ALL

**Figure 2:**
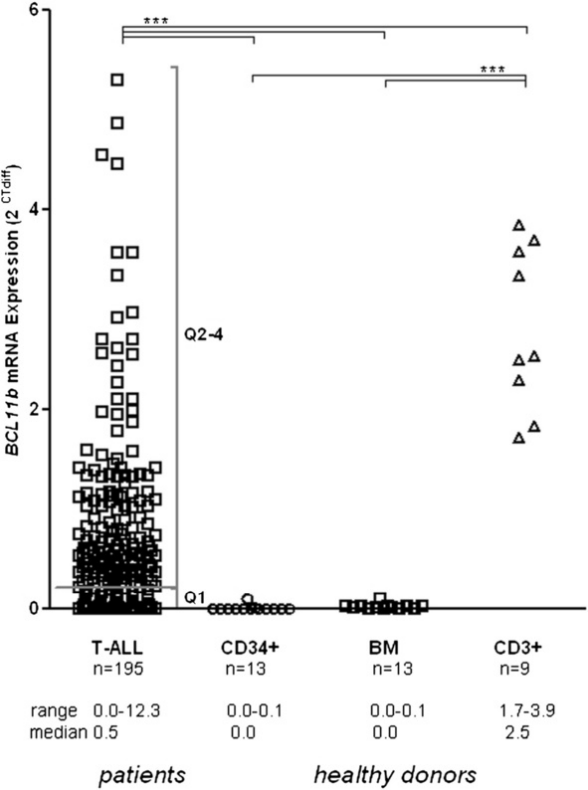
***BCL11b*mRNA expression in T-ALL patients and healthy donors.** A heterogeneous expression pattern was observed within the T-ALL patient samples. Samples from healthy donors had a significantly higher expression of *BCL11b* in CD3 positive mature T-cells than in CD34 positive progenitor cells or unselected bone marrow (BM) samples. T-ALL patients were split into *BCL11b* expression quartiles and patients with low or lacking expression were combined in Q1 and patients with high expression in Q2-4. Outliers (n = 4 T-ALL patients, *BCL11b* expression values: 6.3, 7.6, 9.7, 12.3) were omitted from the diagram but included in the statistical analysis. BM = bone marrow; *** P < 0.01.

*BCL11b* mRNA expression levels were not detectable in CD34 positive hematopoietic progenitor cells or unselected bone marrow (BM) samples from healthy donors, whereas high expression levels were found in CD3 positive mature T-cells (median: 2.5, Figure 2). In contrast, diagnostic BM samples of adult T-ALL patients (n = 195) showed an aberrant and highly heterogeneously *BCL11b* expression pattern (median: 0.5 range = 0-12.3; Figure 2).

As explorative approach, we divided the samples into *BCL11b*-low and *BCL11b*-high expression groups by quartiles. Expression levels in the lowest quartile Q1 (≤0.2) were regarded as *BCL11b*-low, and samples with levels in quartiles Q2-4 (>0.2) as *BCL11b*-high.

### *BCL11b* associated global gene expression profile

**Figure 3:**
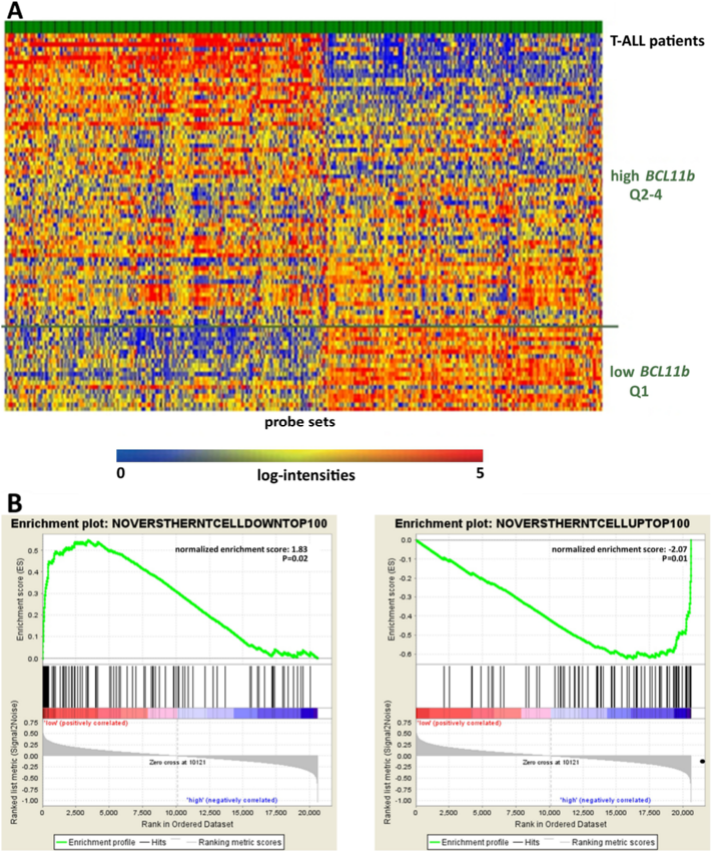
**Gene expression profiles reveal an immature phenotype in*BCL11b*-*low*group. A**: Heat map of two-fold or greater differentially expressed genes between *BCL11b* quartile 1 (Q1) and quartiles 2-4 (Q2-4). **B**: Gene set enrichment analysis for differentially regulated genes in physiological T-cell development according to their *BCL11b* expression. On the left side the top hundred down-regulated genes are shown, on the right side the top hundred up-regulated genes. The lists were taken from Noversthern et al. [[37]].

To explore the underlying transcriptional profile associated with aberrant *BCL11b* expression in T-ALL, we analyzed microarray expression data of an independent cohort of 86 adult T-ALL patients [[33]]. Samples were classified into a low and a high *BCL11b* expression group as described in the material and methods section. We identified 229 up-regulated probe sets (corresponding to 183 unique genes, hypothetical genes/proteins and open reading frames) and 200 down-regulated probe sets (corresponding to 166 genes, hypothetical genes/proteins and open reading frames) in the *BCL11b-*low group compared to the *BCL11b*-high group (Figure 3A, Additional file 1: Table S2 and Additional file 1: Table S3). In the *BCL11b*-low group, genes reported to be suppressed by *BCL11b* were highly expressed: cyclin-dependent kinase inhibitor 1A (p21) and cyclin-dependent kinase inhibitor 1C (p57) [[15],[17]]. Interestingly, genes associated with an immature stem cell-like phenotype were up-regulated in the *BCL11b*-low cohort including *IGFBP7*, *BAALC, CD34,* and *FLT3* [[34]–[36]]. In contrast, the *BCL11b*-high group showed co-expression of markers of a mature T-cell phenotype including several T-cell receptor genes as well as CD8 and CD6. This was further underscored in gene set enrichment analysis (GSEA), in which gene sets associated with physiological T-cell development were enriched in *BCL11b*-high (P < 0.01) and genes down regulated in normal T-cells were enriched in *BCL11b*-low group (P = 0.01; Figure 3B) [[37]].

### *BCL11b* expression with respect to molecular and clinical characteristics

We further explored molecular characteristics associated with *BCL11b* expression in the T-ALL GMALL patient cohort by quantitative RT-PCR. Samples in the lowest expression quartile (Q1; n = 49; *BCL11b* expression range = 0-0.2) were compared to samples with aberrantly high *BCL11b* expression levels defined as Q2-4 (n = 146; *BCL11b* expression range >0.2-12.3, Additional file 1: Table S4). In concordance with the gene expression profiles (GEP) data, these molecular studies by RT-PCR confirmed *IGFBP7* to be overexpressed in the *BCL11b*-low compared to the *BCL11b*-high group. No significant difference between *BCL11b-*low and *BCL11b-*high patients was observed for the previously described negative prognostic factors including *BAALC, ERG*, and *WT1* [[34],[38]]. *GATA3*, a transcription factor up-regulated in DN1 phase of normal T-cell development [[39]] was significantly lower expressed in *BCL11b-*low (median: 2.1 vs. 5.7, P < 0.01) compared to *BCL11b*-high patients. The frequency of TCR rearrangements was significantly lower (50% vs. 80%, P = 0.01) in *BCL11b-*low compared to the *BCL11b*-high group. The *BCL11b* low and the high expressing groups did not differ regarding *NOTCH1* or *WT1* mutation frequencies (Additional file 1: Table S4).

There was no difference with respect to age or sex of T-ALL patients enrolled on the GMALL 06/99 and 07/03 trials, but within the *BCL11b*-low group significantly more patients had a low white blood cell count (WBC; 62% vs. 18% <30.000 × 10^9^/l WBC; P = 0.01; Table 1). Patients with early T-ALL showed a significantly lower *BCL11b* expression (median: 0.3) compared to patients with mature (median: 0.6, P = 0.03) or thymic (median: 0.6, P = 0.01) T-ALL (Additional file 1: Figure S1).

**Table 1:** **Clinical characteristics of GMALL T-ALL patients with respect to** ***BCL11b*** **expression**

**Characteristics**	***BCL11b*-low**	***BCL11b-*** **high**	
	**Q1**	**Q2-4**	**P-value**
n	40	129	
*BCL11b* expression			
Median	0.1	0.8	
Range	0-0.2	0.2-12.3	
Age			n.s.
15-35 yrs	27	70	
36-55 yrs	13	48	
56-65 yrs	0	11	
Sex			n.s.
Female %	20%	26%	
WBC, × 10^9^/l			0.01
<30,000	24	42	
30-100,000	10	53	
>100,000	5	30	
T-ALL subtype			0.01
Early n (%)	17 (42%)	23 (18%)	
Mature n (%)	5 (13%)	22 (17%)	
Thymic n (%)	18 (45%)	84 (65%)	
Response to induction therapy			n.s.
CR n (%)	37 (95%)	115 (94%)	
ED n (%)	1 (3%)	3 (2%)	
Failure n (%)	1 (3%)	5 (4%)	

Abbreviations: *CR* = complete remission, *ED* = early death, *WBC* = white blood cell count.

### *BCL11b* expression is associated with outcome in adult T-ALL

**Figure 4:**
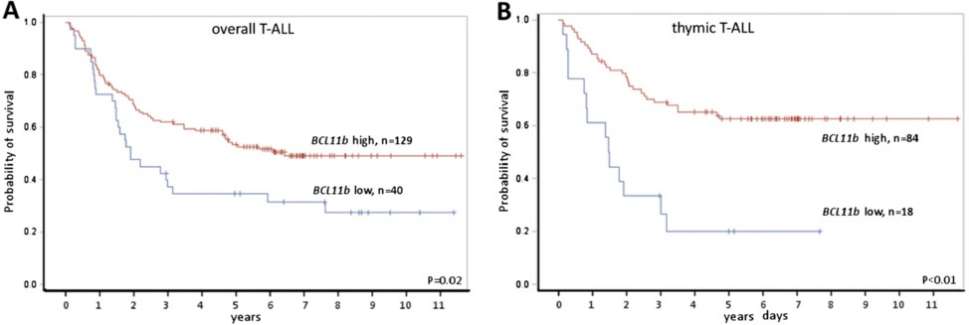
**Kaplan–Meier analyses of overall survival (OS) and remission duration in T-ALL with respect to*BCL11b*mRNA expression. A**: T-ALL patients with low *BCL11b* expression showed significant inferior OS than patients with higher expression (low expression quartile Q1 vs. high Q2-4 expression quartiles; P = 0.02; log-rank test). **B**: The *BCL11b* low expressing thymic T-ALL patients had a highly significant inferior OS (P < 0.01).

While there was no difference between *BCL11b*-low and *BCL11b*-high within the overall GMALL cohort of T-ALL patients with respect to the response to induction therapy (Table 1), the overall survival probability of *BCL11b*-low patients was significantly lower compared to *BCL11b*-high patients (Q1: n = 40, 35% at 5 yrs; Q2-4: n = 129, 53% at 5 yrs; P = 0.02; Figure 4A). When outcomes were analyzed separately for each quartile, T-ALL patients with *BCL11b-*low (Q1) showed an inferior outcome (Q1: n = 40; 35% OS at 5 yrs) compared to patients in the remaining quartile groups (Q2: n = 42; 52% OS at 5 yrs; Q3: n = 42; 52% OS at 5 yrs; Q4: n = 45; 55% OS at 5 yrs; Additional file 1: Figure S2A).

### Low *BCL11b* defines high risk patients within the standard risk group of thymic T-ALL

The identification of novel prognostic markers is in particular important for the largest subgroup of standard risk thymic T-ALL. As these patients are regarded as standard risk, allogeneic stem cell transplantation is not recommended in first complete remission within GMALL trials. In thymic T-ALL, *BCL11b* expression groups did not differ in the expression levels of molecular markers including *BAALC, ERG*, *IGFBP7* and *WT1*. The *BCL11b-*low group showed significantly lower expression of T-cell regulator *GATA3* compared to *BCL11b*-high (median Q1: 1.6, median Q2-4: 5.1, P < 0.01). Significantly fewer thymic *BCL11b*-low patients remained in continuous complete remission at five years compared to *BCL11b*-high thymic T-ALL patients (at 5 yrs: Q1: n = 15, 38%; Q2-4: n = 78, 72%; P = 0.02; Additional file 1: Figure S2B). Moreover, *BCL11b*-low thymic T-ALL had a significantly inferior OS compared to *BCL11b*-high thymic T-ALL patients: while only 20% (n = 18) of *BCL11b*-low patients were alive at 5 years, the 5 year OS of the *BCL11b*-high group was 62% (n = 84; P < 0.01, Figure 4B). Similar to the entire cohort, patients with thymic T-ALL *BCL11b*-low (Q1) showed a significantly inferior outcome compared to higher expression quartiles (Q1: n = 18, 20% at 5 yrs; Q2 n = 26, 67% at 5 yrs¸ Q3 n = 18, 50% at 5 yrs; Q4 n = 30, 70% at 5 yrs; P = 0.01; Additional file 1: Figure S2C).

### High frequency of *BCL11b* mutations in adult T-ALL

**Figure 5:**
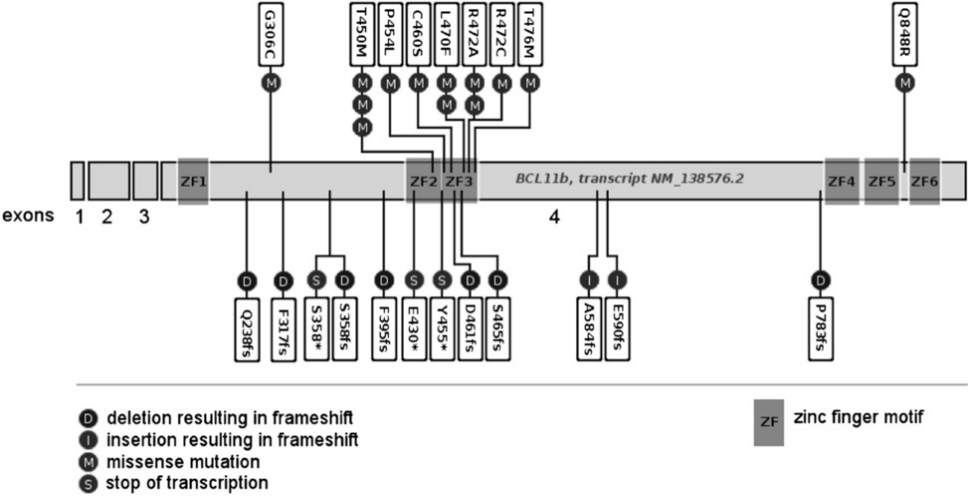
**Mutations found in exon 4 of*BCL11b*in T-ALL.** Mutation analysis was performed by Sanger Sequencing in 178 T-ALL patients, 14% of patients were revealed to harbor protein changing mutations in exon 4 of *BCL11b*.

We sequenced *BCL11b* exon 4 and identified in 14% (24/178) of the T-ALL patients protein modifying alterations (Figure 5, Additional file 1: Table S6). A single T-ALL patient carried two mutations. Sixteen of the mutations were point mutations with single base pair exchanges leading to amino acid exchanges in 13 and a translation stop in three cases. Seven patients carried deletions and two insertions causing frame shifts. 23 of 24 mutations were either located within zinc finger domains or had an impact on these domains through frame shift or stop of translation, and thus have a likely impact on protein function. Nearly all patients with *BCL11b* mutations were in the *BCL11b*-high group (n = 22/23, P = 0.01). The presence of *BCL11b* mutations was associated with the maturation stage of the T-ALL. Of the 21 patients with *BCL11b* mutations, 18 were classified as thymic T-ALL (85.5%; P = 0.03; Additional file 1: Table S7). *WT1*, a negative prognostic factor, was significantly lower expressed in the *BCL11b* mutated group and other known oncogenes or molecular factors were not associated with *BCL11b* mutation status (not shown). However, outcome analyses showed no significant impact of *BCL11b* mutations and deletions on overall survival (Additional file 1: Figure S3).

## Discussion

During normal T-cell development, T-cell progenitors pass through separate differentiation stages and this process is tightly regulated by lineage specific transcription factors. While onset of *GATA3* and *TCF-1* expression characterize the early DN1 (ETP) phase in T-cell differentiation, *BCL11b* is expressed at the later DN2 stage [[39]]. As a gatekeeper of T-cell development, it is essential for the correct αβ T-cell development [[3],[40]]. Loss of *BCL11b* at various stages was shown to induce a natural killer cell-like phenotype with a differentiation arrest and was associated with a high proliferative potential [[14],[40]]. In addition, *BCL11b* is necessary for T-cell identity maintenance. Alteration of different stage-specific transcription factors and key regulators of T-cell differentiation by silencing, ectopic expression or mutations have shown to contribute in oncogenic transformation in T-ALL. For example, aberrant *NOTCH1* signaling through activating mutations found in 50-60% of T-ALL cases [[41],[42]], is a prominent example of a potent driver event in T-cell leukemogenesis. For *TAL1*, which plays a key role in hematopoietic stem cell development, rearrangements and aberrant expression were found in T-ALL patients [[43]]. In this case, *TAL1* expression was positively correlated with survival in pediatric patients [[44]]. Likewise for the homeobox transcription factor *TXL-1* deregulation and gene locus abnormalities had been associated with improved outcome for *TXL*-1-high T-ALL patients [[45]]. Understanding of the molecular processes involved in T-ALL pathology offers the possibility to refine prognosis and stratify therapeutic algorithms.

For *BCL11b* aberrant expression levels, deletions and mutations have been reported in T-ALL [[29]–[32],[46],[47]]. Here we comprehensively investigated the implications of altered *BCL11b* expression and loss of function mutations in a large cohort of adult T-ALL patients (n = 169). While CD34^+^ hematopoietic progenitor cells and unselected BM samples of healthy donors lack *BCL11b* expression, T-ALL patients showed an aberrant and highly heterogonous *BCL11b* expression pattern. Similar to normal T-cell differentiation, the expression of *BCL11b* reflected the maturation stage in T-ALL and thus was significantly lower in the early compared to thymic and mature T-ALL subgroups (Figure 1, Additional file 1: Figure S1). Analysis of microarray expression data confirmed this observation revealing up-regulation of genes associated with an immature phenotype in *BCL11b*-low and an enrichment of markers of a mature T-cell phenotype in *BCL11b*-high T-ALL.

While the combined patient data suggested that *BCL11b* expression reflects the differentiation arrest of leukemic cells, expression was heterogeneously distributed and patients that lacked or had very low *BCL11b* expression were found in all immunophenotypic subgroups. This suggests that *BCL11b* is not just a mere marker of genetically more differentiated blasts, but may act as a maturation dependent tumor suppressor, which is supported in other studies [[29],[30],[47]]. If deregulated during differentiation, disruption of normal *BCL11b* function may contribute to malignant transformation.

In this study the cohort of uniformly treated adult T-ALL patients, the *BCL11b*-low subgroup had a significantly inferior OS compared to the *BCL11b*-high patients. In particular, in the standard risk group of thymic T-ALL, *BCL11b* was of relevant prognostic impact: 62% of *BCL11b*-high patients were alive after 5 years, whereas survival was only 20% at 5 years in the *BCL11b*-low subgroup. Remission duration was also significantly shorter for patients within the *BCL11b-*low subgroup. This contrasts a study in pediatric T-ALL patients, which showed that *BCL11b* expression had no impact on OS [[30]], although the difference may be due to the smaller sample size of the study and that patients were not classified into immunophenotypic subgroups. Nevertheless, low *BCL11b* expression was associated with chemotherapy induction failure in the same study.

While immunophenotypic classification of T-ALL has improved outcome prediction, a relevant percentage of patients classified as standard risk based on their thymic immunophenotype, fail conventional chemotherapy. As thymic T-ALL on the molecular and clinical levels compromises a highly heterogeneous cohort, it remains essential to identify patients with a high risk of relapse to adjust treatment stratification. Our results indicate that thymic *BCL11b*-low T-ALL patients represent a high risk subgroup which would benefit from intensified MRD monitoring and treatment stratification including allogeneic stem cell transplantation. Since lack of *BCL11b* expression proved to indicate inferior survival, we investigated disruption of the gene’s function on the mutational level. *BCL11b* mutations in pediatric and adult patients had been reported in T-ALL in the zinc finger structures of exon 4 [[29],[30],[32],[46]]. In agreement with these studies, we discovered a high rate (14%) of *BCL11b* mutations in this large cohort of T-ALL patients. We found an association of immunophenotype and mutation frequency: only 2% mutational rate in early T-ALL patients and 19% mutational rate in thymic T-ALL patients. Our results support a recent study in which adult T-ALL patients characterized as “early/immature” had fewer *BCL11b* mutations [[32]]. The number of *BCL11b* mutations in thymic T-ALL samples in this report was low, limiting the statistical significance regarding outcome. Also, gene expression studies may be more sensitive to identify patients with the specific outcome-associated phenotype caused by *BCL11b* down-regulation.

Further studies are needed to fully understand the biological relevance of *BCL11b* mutations, and to explore potential directed therapies.

In summary, we identified *BCL11b* expression as a potent prognostic factor in the overall cohort and in particular in the standard risk subgroup of thymic T-ALL. These findings will help to identify patients with an enhanced risk of failure to standard therapy, however, standardized detection analyses of aberrant gene expression levels in a routine diagnostic setting remains challenging. More importantly, alterations in critical transcription factors contribute to leukemogenesis and may be regarded as ideal candidates for differentiation directed therapies in the future.

## Methods

### Patients

We analyzed diagnostic BM material of 195 adult T-ALL patients sent to the GMALL reference laboratory [[48]]. Immunophenotyping of the samples was centrally performed in the GMALL reference laboratory at the Charité, University Hospital Berlin, Germany, as previously described [[49],[50]], and classified into the T-ALL subgroups: early (n = 50), mature (n = 33) and thymic (n = 112). Of these, 169 T-ALL patients were consecutively enrolled in the GMALL trials 06/99 and 07/2003 [[51]] with available clinical follow-up data. Additionally, samples of healthy adult donors were used, 13 BM samples, 13 CD34+ progenitors and nine CD3+ selected T-cell samples. Written informed consent according to the declaration of Helsinki had been given. Studies were approved by the ethics board and registered in a public registry (clincaltrials.gov NCT00199056, NCT0098991).

### Sample preparation and qRT-PCR

Total RNA and DNA extractions were performed using TRIzol reagent (Invitrogen) according to the manufacturer’s instructions. To analyze *BCL11b* expression, complementary DNA was synthesized and quantitative real-time polymerase chain reaction (qRT-PCR) was performed, using *Glucose-6-Phosphate Isomerase* (*GPI*) as internal control, as previously described [[52]]. For *BCL11b* amplification forward primer AACCCGCAGCACTTGTCC, reverse-primer ATTTGACACTGGCCACAGGT and probe FAM-CTCATCACCCCAGAGGCTGACCAT-BHQ1 spanning exons 1 and 2 were used. Expression levels of *BCL11b* were calculated using the mean of ΔCT from two replicates and expressed as 2^μ(ΔCT)^. The mRNA expression levels for *IGFBP7*, *WT1*, *BAALC*, *ERG,* and *GATA3* by qRT-PCR, as well as mutation status of *WT1* and *NOTCH1* and TCR-rearrangements had been determined in previous studies [[12],[34],[35],[52]–[54]].

### Gene expression profiles

*BCL11b*-associated GEP of an independent set of 86 adult T-ALL samples were generated from raw data obtained from the Microarrays Innovations in Leukemia (MILE) multicenter study (HG-U133 Plus 2.0; Affymetrix, Santa Clara, CA, USA) [[33]]. For the GEP-analysis, samples were divided into quartiles (Q) according to *BCL11b* expression [median of the two probe sets (219528_s_at, 222895_s_at)]. To identify *BCL11b*-associated GEP signatures, the lowest expression quartile (Q1) was compared to quartiles 2 to 4 united (Q2-4). Lists of genes with a 2-fold under- or over-expression were generated. Statistical significance was calculated by the non-parametric *t*-test with a *P*-value ≤0.05. The data analyses were carried out with GeneSpring software version 4.2 (Silicon Genetics, Redwood City, CA, USA).

### Mutational analysis

Quantity and quality of genomic DNA was sufficient for the mutational analysis of *BCL11b* in 178 T-ALL samples. As previous studies had detected only very few mutations outside exon 4, which harbors all six of the gene’s zinc finger domains, we focused on this region [[29],[30]]. Primer pairs were newly designed or used as previously published for bidirectional Sanger sequencing of exon 4 (complete list see Additional file 1: Table S1) [[29]]. Geneious version 5.4.3 software (Biomatters Ltd., Auckland, NZ) was used for analysis.

## Abbreviations

ALL: Acute lymphoblastic leukemia

AML: Acute myeloid leukemia

BM: Bone marrow

DN: Double negative

ETP-ALL: Early t-cell precursor ALL

GEP: Gene expression profile

GSEA: Gene set enrichment analysis

GMALL: German multicenter acute lymphoblastic leukemia study group

MILE: Microarrays innovations in leukemia

MRD: Minimal residual disease

OS: Overall survival

qRT-PCR: Quantitative real-time polymerase chain reaction

T-ALL: T-cell acute lymphoblastic leukemia

TKI: Tyrosine kinase inhibitors

## Competing interests

The authors declare that they have no competing interests.

## Authors’ contributions

IB performed laboratory work, data analysis and wrote the manuscript. NG supervised the GMALL study center, performed statistical analysis and reviewed the manuscript. CS performed laboratory work for this study. SH helped to design the study. LF provided expression data. SS, RS, KSE, MS, AR and DH recruited the study patients and performed the study procedures. CDB coordinate the research and reviewed the manuscript. MN performed statistical analysis and reviewed the manuscript. All authors read and approved the final manuscript.

## Additional file

## Supplementary Material

Additional file 1: **Statistical analysis. Figure S1.** BCL11b mRNA expression in T-ALL immunophenotypic subtypes. **Figure S2.** Kaplan–Meier analysis of overall survival (OS) in T-ALL with respect to BCL11b mRNA expression. **Figure S3.** Kaplan–Meier analysis of overall survival (OS) in T-ALL with respect to BCL11b mutation status. **Table S1.** Primer sets designed for human BCL11b exon 4 (NP_612808.1). **Table S2.** Probe sets up-regulated in the BCL11b-low group of T-ALL patients. **Table S3.** Probe sets up-regulated in the BCL11b-high group of T-ALL patients. **Table S4.** Molecular characteristics of T-ALL patients with respect to BCL11b expression. **Table S5.** Molecular characteristics of thymic GMALL T-ALL patients in respect to BCL11b expression. **Table S6.** BCL11b exon 4 mutations in T-ALL. **Table S7.** Clinical characteristics of GMALL T-ALL patients in respect to BCL11b mutations. **Table S8.** Members of the German Multicenter Study Group for Adult ALL Supplemental Methods: Statistics. References. Click here for file

## References

[B1] Gokbuget N Hoelzer D Treatment of adult acute lymphoblastic leukemia Semin Hematol 2009 46 64 75 10.1053/j.seminhematol.2008.09.003 19100369

[B2] Hunault M Harousseau JL Delain M Truchan-Graczyk M Cahn JY Witz F Lamy T Pignon B Jouet JP Garidi R Caillot D Berthou C Guyotat D Sadoun A Sotto JJ Lioure B Casassus P Solal-Celigny P Stalnikiewicz L Audhuy B Blanchet O Baranger L Béné MC Ifrah N Better outcome of adult acute lymphoblastic leukemia after early genoidentical allogeneic bone marrow transplantation (BMT) than after late high-dose therapy and autologous BMT: a GOELAMS trial Blood 2004 104 3028 3037 10.1182/blood-2003-10-3560 15256423

[B3] Bassan R Hoelzer D Modern therapy of acute lymphoblastic leukemia J Clin Oncol 2011 29 532 543 10.1200/JCO.2010.30.1382 21220592

[B4] Hoelzer D Novel antibody-based therapies for acute lymphoblastic leukemia Hematol Am Soc Hematol Educ Progr 2011 2011 243 249 10.1182/asheducation-2011.1.243 22160041

[B5] Daver N O'Brien S Novel therapeutic strategies in adult acute lymphoblastic leukemia - a focus on emerging monoclonal antibodies Curr Hematol Malig Rep 2013 8 123 131 10.1007/s11899-013-0160-7 23539383 PMC4438701

[B6] Topp MS Gokbuget N Zugmaier G Degenhard E Goebeler ME Klinger M Neumann SA Horst HA Raff T Viardot A Stelljes M Schaich M Köhne-Volland R Brüggemann M Ottmann OG Burmeister T Baeuerle PA Nagorsen D Schmidt M Einsele H Riethmüller G Kneba M Hoelzer D Kufer P Bargou RC Long-term follow-up of hematologic relapse-free survival in a phase 2 study of blinatumomab in patients with MRD in B-lineage ALL Blood 2012 120 5185 5187 10.1182/blood-2012-07-441030 23024237

[B7] Berg SL Blaney SM Devidas M Lampkin TA Murgo A Bernstein M Billett A Kurtzberg J Reaman G Gaynon P Whitlock J Krailo M Harris MB Phase II study of nelarabine (compound 506U78) in children and young adults with refractory T-cell malignancies: a report from the children’s oncology group J Clin Oncol 2005 23 3376 3382 10.1200/JCO.2005.03.426 15908649

[B8] Robak T New nucleoside analogs for patients with hematological malignancies Expert Opin Investig Drugs 2011 20 343 359 10.1517/13543784.2011.554822 21320002

[B9] Palomero T Ferrando A Therapeutic targeting of NOTCH1 signaling in T-cell acute lymphoblastic leukemia Clin Lymphoma Myeloma 2009 9 Suppl 3 S205 S210 10.3816/CLM.2009.s.013 19778842 PMC2814179

[B10] Coustan-Smith E Mullighan CG Onciu M Behm FG Raimondi SC Pei D Cheng C Su X Rubnitz JE Basso G Biondi A Pui CH Downing JR Campana D Early T-cell precursor leukaemia: a subtype of very high-risk acute lymphoblastic leukaemia Lancet Oncol 2009 10 147 156 10.1016/S1470-2045(08)70314-0 19147408 PMC2840241

[B11] Meijerink JP Genetic rearrangements in relation to immunophenotype and outcome in T-cell acute lymphoblastic leukaemia Best Pract Res Clin Haematol 2010 23 307 318 10.1016/j.beha.2010.08.002 21112032

[B12] Neumann M, Heesch S, Gokbuget N, Schwartz S, Schlee C, Benlasfer O, Farhadi-Sartangi N, Thibaut J, Burmeister T, Hoelzer D, Hofmann WK, Thiel E, Baldus CD: **Clinical and molecular characterization of early T-cell precursor leukemia: a high-risk subgroup in adult T-ALL with a high frequency of FLT3 mutations.** *Blood Cancer J* 2012, **2:**e55. 10.1038/bcj.2011.49 PMC3270253 22829239

[B13] Liu P Li P Burke S Critical roles of Bcl11b in T-cell development and maintenance of T-cell identity Immunol Rev 2010 238 138 149 10.1111/j.1600-065X.2010.00953.x 20969590

[B14] Kominami R Role of the transcription factor Bcl11b in development and lymphomagenesis Proc Jpn Acad Ser B Phys Biol Sci 2012 88 72 87 10.2183/pjab.88.72 22450536 PMC3365246

[B15] Cherrier T Suzanne S Redel L Calao M Marban C Samah B Mukerjee R Schwartz C Gras G Sawaya BE Zeichner SL Aunis D Van Lint C Rohr O p21(WAF1) gene promoter is epigenetically silenced by CTIP2 and SUV39H1 Oncogene 2009 28 3380 3389 10.1038/onc.2009.193 19581932 PMC3438893

[B16] Cismasiu VB Ghanta S Duque J Albu DI Chen HM Kasturi R Avram D BCL11B participates in the activation of IL2 gene expression in CD4+ T lymphocytes Blood 2006 108 2695 2702 10.1182/blood-2006-05-021790 16809611 PMC1895584

[B17] Topark-Ngarm A Golonzhka O Peterson VJ Barrett B Jr Martinez B Crofoot K Filtz TM Leid M CTIP2 associates with the NuRD complex on the promoter of p57KIP2, a newly identified CTIP2 target gene J Biol Chem 2006 281 32272 32283 10.1074/jbc.M602776200 16950772 PMC2547407

[B18] Grabarczyk P Przybylski GK Depke M Volker U Bahr J Assmus K Broker BM Walther R Schmidt CA Inhibition of BCL11B expression leads to apoptosis of malignant but not normal mature T cells Oncogene 2007 26 3797 3810 10.1038/sj.onc.1210152 17173069

[B19] Karanam NK Grabarczyk P Hammer E Scharf C Venz S Gesell-Salazar M Barthlen W Przybylski GK Schmidt CA Volker U Proteome analysis reveals new mechanisms of Bcl11b-loss driven apoptosis J Proteome Res 2010 9 3799 3811 10.1021/pr901096u 20513151

[B20] Grabarczyk P, Nahse V, Delin M, Przybylski G, Depke M, Hildebrandt P, Volker U, Schmidt CA: **Increased expression of bcl11b leads to chemoresistance accompanied by G1 accumulation.** *PLoS One* 2010, **5:**e12532. 10.1371/journal.pone.0012532 PMC2932720 20824091

[B21] Wakabayashi Y Inoue J Takahashi Y Matsuki A Kosugi-Okano H Shinbo T Mishima Y Niwa O Kominami R Homozygous deletions and point mutations of the Rit1/Bcl11b gene in gamma-ray induced mouse thymic lymphomas Biochem Biophys Res Commun 2003 301 598 603 10.1016/S0006-291X(02)03069-3 12565905

[B22] Kamimura K Ohi H Kubota T Okazuka K Yoshikai Y Wakabayashi Y Aoyagi Y Mishima Y Kominami R Haploinsufficiency of Bcl11b for suppression of lymphomagenesis and thymocyte development Biochem Biophys Res Commun 2007 355 538 542 10.1016/j.bbrc.2007.02.003 17306224

[B23] MacLeod RA Nagel S Kaufmann M Janssen JW Drexler HG Activation of HOX11L2 by juxtaposition with 3′-BCL11B in an acute lymphoblastic leukemia cell line (HPB-ALL) with t(5;14)(q35;q32.2) Genes Chromosomes Cancer 2003 37 84 91 10.1002/gcc.10194 12661009

[B24] Przybylski GK Dik WA Wanzeck J Grabarczyk P Majunke S Martin-Subero JI Siebert R Dolken G Ludwig WD Verhaaf B van Dongen JJ Schmidt CA Langerak AW Disruption of the BCL11B gene through inv(14)(q11.2q32.31) results in the expression of BCL11B-TRDC fusion transcripts and is associated with the absence of wild-type BCL11B transcripts in T-ALL Leukemia 2005 19 201 208 10.1038/sj.leu.2403619 15668700

[B25] Satterwhite E Sonoki T Willis TG Harder L Nowak R Arriola EL Liu H Price HP Gesk S Steinemann D Schlegelberger B Oscier DG Siebert R Tucker PW Dyer MJ The BCL11 gene family: involvement of BCL11A in lymphoid malignancies Blood 2001 98 3413 3420 10.1182/blood.V98.12.3413 11719382

[B26] Bezrookove V van Zelderen-Bhola SL Brink A Szuhai K Raap AK Barge R Beverstock GC Rosenberg C A novel t(6;14)(q25-q27;q32) in acute myelocytic leukemia involves the BCL11B gene Cancer Genet Cytogenet 2004 149 72 76 10.1016/S0165-4608(03)00302-9 15104287

[B27] Oliveira JL Kumar R Khan SP Law ME Erickson-Johnson M Oliveira AM Ketterling RP Dogan A Successful treatment of a child with T/myeloid acute bilineal leukemia associated with TLX3/BCL11B fusion and 9q deletion Pediatr Blood Cancer 2011 56 467 469 10.1002/pbc.22850 21225930

[B28] Abbas S Sanders M Zeilemaker A Geertsma-Kleinekoort WM Koenders JE Kavelaars FG Abbas ZG Mahamoud S Chu IW Hoogenboezem R Peeters JK van Drunen E van Galen J Beverloo HB Löwenberg B Valk PJ Integrated genome-wide genotyping and gene expression profiling reveals BCL11B as a putative oncogene in acute myeloid leukemia with 14q32 aberrations Haematologica 2014 99 848 57 10.3324/haematol.2013.095604 24441149 PMC4008111

[B29] De Keersmaecker K Real PJ Gatta GD Palomero T Sulis ML Tosello V Van VP Barnes K Castillo M Sole X Hadler M Lenz J Aplan PD Kelliher M Kee BL Pandolfi PP Kappes D Gounari F Petrie H Van der Meulen J Speleman F Paietta E Racevskis J Wiernik PH Rowe JM Soulier J Avran D Cavé H Dastugue N Raimondi S The TLX1 oncogene drives aneuploidy in T cell transformation Nat Med 2010 16 1321 1327 10.1038/nm.2246 20972433 PMC2974790

[B30] Gutierrez A Kentsis A Sanda T Holmfeldt L Chen SC Zhang J Protopopov A Chin L Dahlberg SE Neuberg DS Silverman LB Winter SS Hunger SP Sallan SE Zha S Alt FW Downing JR Mullighan CG Look AT The BCL11B tumor suppressor is mutated across the major molecular subtypes of T-cell acute lymphoblastic leukemia Blood 2011 118 4169 4173 10.1182/blood-2010-11-318873 21878675 PMC3204734

[B31] De Keersmaecker K Atak ZK Li N Vicente C Patchett S Girardi T Gianfelici V Geerdens E Clappier E Porcu M Lahortiga I Lucà R Yan J Hulselmans G Vranckx H Vandepoel R Sweron B Jacobs K Mentens N Wlodarska I Cauwelier B Cloos J Soulier J Uyttebroeck A Bagni C Hassan BA Vandenberghe P Johnson AW Aerts S Cools J Exome sequencing identifies mutation in CNOT3 and ribosomal genes RPL5 and RPL10 in T-cell acute lymphoblastic leukemia Nat Genet 2013 45 186 190 10.1038/ng.2508 23263491 PMC5547913

[B32] Van Vlierberghe P Ambesi-Impiombato A De Keersmaecker K Hadler M Paietta E Tallman MS Rowe JM Forne C Rue M Ferrando AA Prognostic relevance of integrated genetic profiling in adult T-cell acute lymphoblastic leukemia Blood 2013 122 74 82 10.1182/blood-2013-03-491092 23687089 PMC3701905

[B33] Haferlach T Kohlmann A Wieczorek L Basso G Kronnie GT Bene MC De VJ Hernandez JM Hofmann WK Mills KI Gilkes A Chiaretti S Shurtleff SA Kipps TJ Rassenti LZ Yeoh AE Papenhausen PR Liu WM Williams PM Foà R Clinical utility of microarray-based gene expression profiling in the diagnosis and subclassification of leukemia: report from the international microarray innovations in leukemia study group J Clin Oncol 2010 28 2529 2537 10.1200/JCO.2009.23.4732 20406941 PMC5569671

[B34] Baldus CD Martus P Burmeister T Schwartz S Gokbuget N Bloomfield CD Hoelzer D Thiel E Hofmann WK Low ERG and BAALC expression identifies a new subgroup of adult acute T-lymphoblastic leukemia with a highly favorable outcome J Clin Oncol 2007 25 3739 3745 10.1200/JCO.2007.11.5253 17646667

[B35] Heesch S Schlee C Neumann M Stroux A Kuhnl A Schwartz S Haferlach T Goekbuget N Hoelzer D Thiel E Hofmann WK Baldus CD BAALC-associated gene expression profiles define IGFBP7 as a novel molecular marker in acute leukemia Leukemia 2010 24 1429 1436 10.1038/leu.2010.130 20535151

[B36] Small D Levenstein M Kim E Carow C Amin S Rockwell P Witte L Burrow C Ratajczak MZ Gewirtz AM Civin CI STK-1, the human homolog of Flk-2/Flt-3, is selectively expressed in CD34+ human bone marrow cells and is involved in the proliferation of early progenitor/stem cells Proc Natl Acad Sci U S A 1994 91 459 463 10.1073/pnas.91.2.459 7507245 PMC42968

[B37] Novershtern N Subramanian A Lawton LN Mak RH Haining WN McConkey ME Habib N Yosef N Chang CY Shay T Frampton GM Drake AC Leskov I Nilsson B Preffer F Dombkowski D Evans JW Liefeld T Smutko JS Chen J Friedman N Young RA Golub TR Regev A Ebert BL Densely interconnected transcriptional circuits control cell states in human hematopoiesis Cell 2011 144 296 309 10.1016/j.cell.2011.01.004 21241896 PMC3049864

[B38] Menssen HD Renkl HJ Rodeck U Maurer J Notter M Schwartz S Reinhardt R Thiel E Presence of Wilms' tumor gene (wt1) transcripts and the WT1 nuclear protein in the majority of human acute leukemias Leukemia 1995 9 1060 1067 7596170

[B39] Rothenberg EV Transcriptional drivers of the T-cell lineage program Curr Opin Immunol 2012 24 132 138 10.1016/j.coi.2011.12.012 22264928 PMC3319509

[B40] Li L Leid M Rothenberg EV An early T cell lineage commitment checkpoint dependent on the transcription factor Bcl11b Science 2010 329 89 93 10.1126/science.1188989 20595614 PMC2935300

[B41] Koch U Radtke F Notch in T-ALL: new players in a complex disease Trends Immunol 2011 32 434 442 10.1016/j.it.2011.06.005 21775206

[B42] Weng AP Ferrando AA Lee W Morris JP Silverman LB Sanchez-Irizarry C Blacklow SC Look AT Aster JC Activating mutations of NOTCH1 in human T cell acute lymphoblastic leukemia Science 2004 306 269 271 10.1126/science.1102160 15472075

[B43] Van VP Ferrando A The molecular basis of T cell acute lymphoblastic leukemia J Clin Invest 2012 122 3398 3406 10.1172/JCI61269 23023710 PMC3461904

[B44] Kikuchi A Hayashi Y Kobayashi S Hanada R Moriwaki K Yamamoto K Fujimoto J Kaneko Y Yamamori S Clinical significance of TAL1 gene alteration in childhood T-cell acute lymphoblastic leukemia and lymphoma Leukemia 1993 7 933 938 8321044

[B45] Bergeron J Clappier E Radford I Buzyn A Millien C Soler G Ballerini P Thomas X Soulier J Dombret H Macintyre EA Asnafi V Prognostic and oncogenic relevance of TLX1/HOX11 expression level in T-ALLs Blood 2007 110 2324 2330 10.1182/blood-2007-04-079988 17609427

[B46] Kraszewska MD Dawidowska M Kosmalska M Sedek L Grzeszczak W Kowalczyk JR Szczepanski T Witt M BCL11B, FLT3, NOTCH1 and FBXW7 mutation status in T-cell acute lymphoblastic leukemia patients Blood Cells Mol Dis 2013 50 33 38 10.1016/j.bcmd.2012.09.001 23040356

[B47] Kurosawa N, Fujimoto R, Ozawa T, Itoyama T, Sadamori N, Isobe M: **Reduced level of the BCL11B protein is associated with adult T-cell leukemia/lymphoma.** *PLoS One* 2013, **8:**e55147. 10.1371/journal.pone.0055147 PMC3559337 23383087

[B48] Brüggemann M Raff T Flohr T Gokbuget N Nakao M Droese J Luschen S Pott C Ritgen M Scheuring U Horst HA Thiel E Hoelzer D Bartram CR Kneba M Clinical significance of minimal residual disease quantification in adult patients with standard-risk acute lymphoblastic leukemia Blood 2006 107 1116 1123 10.1182/blood-2005-07-2708 16195338

[B49] Baldus CD Thibaut J Goekbuget N Stroux A Schlee C Mossner M Burmeister T Schwartz S Bloomfield CD Hoelzer D Thiel E Hofmann WK Prognostic implications of NOTCH1 and FBXW7 mutations in adult acute T-lymphoblastic leukemia Haematologica 2009 94 1383 1390 10.3324/haematol.2008.005272 19794083 PMC2754954

[B50] Gokbuget N Raff R Brüggemann M Flohr T Scheuring U Pfeifer H Bartram CR Kneba M Hoelzer D Risk/MRD adapted GMALL trials in adult ALL Ann Hematol 2004 83 Suppl 1 S129 S131 15124705 10.1007/s00277-004-0850-2

[B51] http://www.leukemia-net.org/trial/download/public/ALL_GMALL07-03_ShortProtEN.pdf?id=489 **European leukemia Net. European leukemia trial registry trial: ALL GMALL 07/2003.**. Accessed September 3, 2013. .

[B52] Baldus CD Burmeister T Martus P Schwartz S Gokbuget N Bloomfield CD Hoelzer D Thiel E Hofmann WK High expression of the ETS transcription factor ERG predicts adverse outcome in acute T-lymphoblastic leukemia in adults J Clin Oncol 2006 24 4714 4720 10.1200/JCO.2006.06.1580 16954520

[B53] Heesch S Goekbuget N Stroux A Tanchez JO Schlee C Burmeister T Schwartz S Blau O Keilholz U Busse A Hoelzer D Thiel E Hofmann WK Baldus CD Prognostic implications of mutations and expression of the Wilms tumor 1 (WT1) gene in adult acute T-lymphoblastic leukemia Haematologica 2010 95 942 949 10.3324/haematol.2009.016386 20435628 PMC2878792

[B54] Neumann M, Coskun E, Fransecky L, Mochmann LH, Bartram I, Sartangi NF, Heesch S, Gokbuget N, Schwartz S, Brandts C, Schlee C, Haas R, Dührsen U, Griesshammer M, Döhner H, Ehninger G, Burmeister T, Blau O, Thiel E, Hoelzer D, Hofmann WK, Baldus CD: **FLT3 mutations in early T-cell precursor ALL characterize a stem cell like leukemia and imply the clinical use of tyrosine kinase inhibitors.** *PLoS One* 2013, **8:**e53190. 10.1371/journal.pone.0053190 PMC3554732 23359050

