# Supplementary material for: Low expression of T-cell transcription factor BCL11b predicts inferior survival in adult standard risk T-cell acute lymphoblastic leukemia patients
Source: J Hematol Oncol. 2014 Jul 15;7:51. doi: 10.1186/s13045-014-0051-y (PMC4223626; doi:10.1186/s13045-014-0051-y)
Supplement: Additional file 1: — Statistical analysis. Figure S1. BCL11b mRNA expression in T-ALL immunophenotypic subtypes. Figure S2. Kaplan–Meier analysis of overall survival (OS) in T-ALL with respect to BCL11b mRNA expression. Figure S3. Kaplan–Meier analysis of overall survival (OS) in T-ALL with respect to BCL11b mutation status. Table S1. Primer sets designed for human BCL11b exon 4 (NP_612808.1). Table S2. Probe sets up-regulated in the BCL11b-low group of T-ALL patients. Table S3. Probe sets up-regulated in the BCL11b-high group of T-ALL patients. Table S4. Molecular characteristics of T-ALL patients with respect to BCL11b expression. Table S5. Molecular characteristics of thymic GMALL T-ALL patients in respect to BCL11b expression. Table S6. BCL11b exon 4 mutations in T-ALL. Table S7. Clinical characteristics of GMALL T-ALL patients in respect to BCL11b mutations. Table S8. Members of the German Multicenter Study Group for Adult ALL Supplemental Methods: Statistics. References. [file s13045-014-0051-y-S1.pdf]

**Supplement for Bartram *et al.* 2014**

**<u>Content:</u>**

**Figure S1: *BCL11b* mRNA expression in T-ALL immunophenotypic subtypes**

**Figure S2: Kaplan–Meier analysis of overall survival (OS) in T-ALL with respect to *BCL11b* mRNA expression.**

**Figure S3: Kaplan–Meier analysis of overall survival (OS) in T-ALL with respect to *BCL11b* mutation status.**

**Table S1: Primer sets designed for human *BCL11b* exon 4 (NP_612808.1)**

**Table S2: Probe sets up-regulated in the *BCL11b*-low group of T-ALL patients**

**Table S3: Probe sets up-regulated in the *BCL11b*-high group of T-ALL patients**

**Table S4: Molecular characteristics of T-ALL patients with respect to BCL11b expression**

**Table S5: Molecular characteristics of thymic GMALL T-ALL patients in respect to *BCL11b* expression**

**Table S6: *BCL11b* exon 4 mutations in T-ALL**

**Table S7: Clinical characteristics of GMALL T-ALL patients in respect to *BCL11b* mutations**

**Table S8: Members of the German Multicenter Study Group for Adult ALL**

**Supplemental Methods: Statistics**

**References**

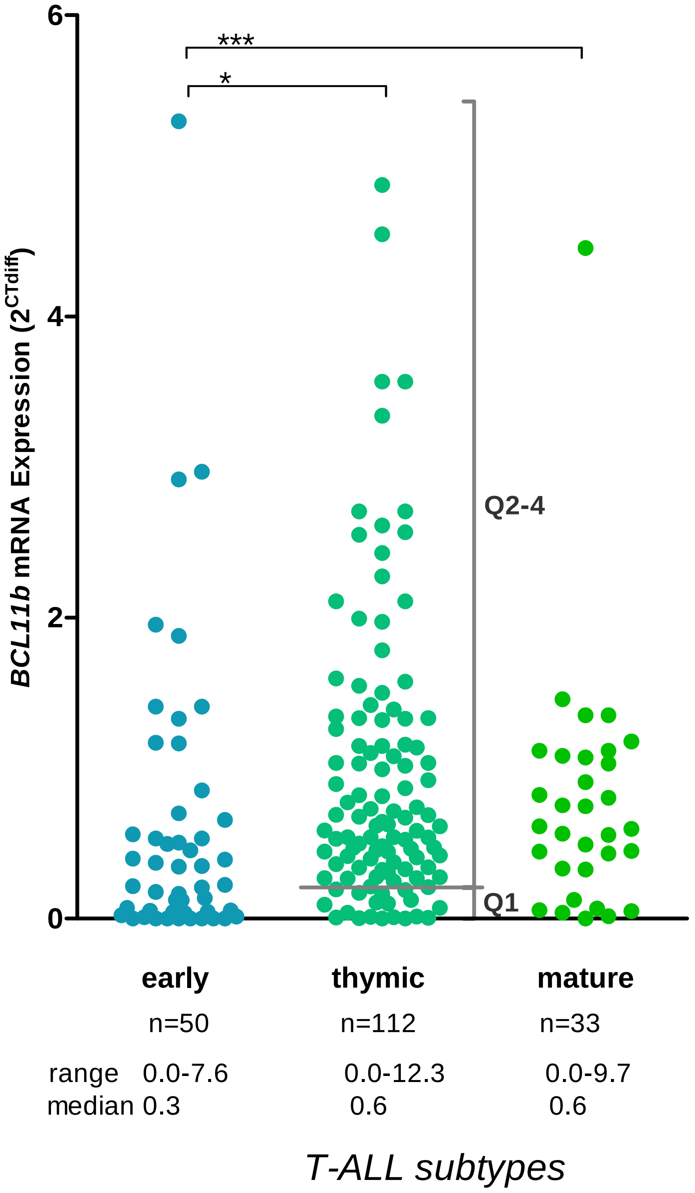


**Figure S1: *BCL11b* mRNA expression in T-ALL immunophenotypic subtypes**
Patients with early T-ALL immunophenotype showed a significantly lower expression than thymic or mature T-ALL patients. *BCL11b* expression quartiles were defined on the basis of the whole T-ALL cohort for further analysis. Patients with low or lacking expression were combined in Q1 and patients with high expression in Q2-4.
Outliers (n=4 thymic T-ALL patients, *BCL11b* expression values: 6.3, 7.6, 9.7, 12.3) were omitted from the diagram but included in the statistical analysis. BM = bone marrow; * P=0.03, *** P<0.01

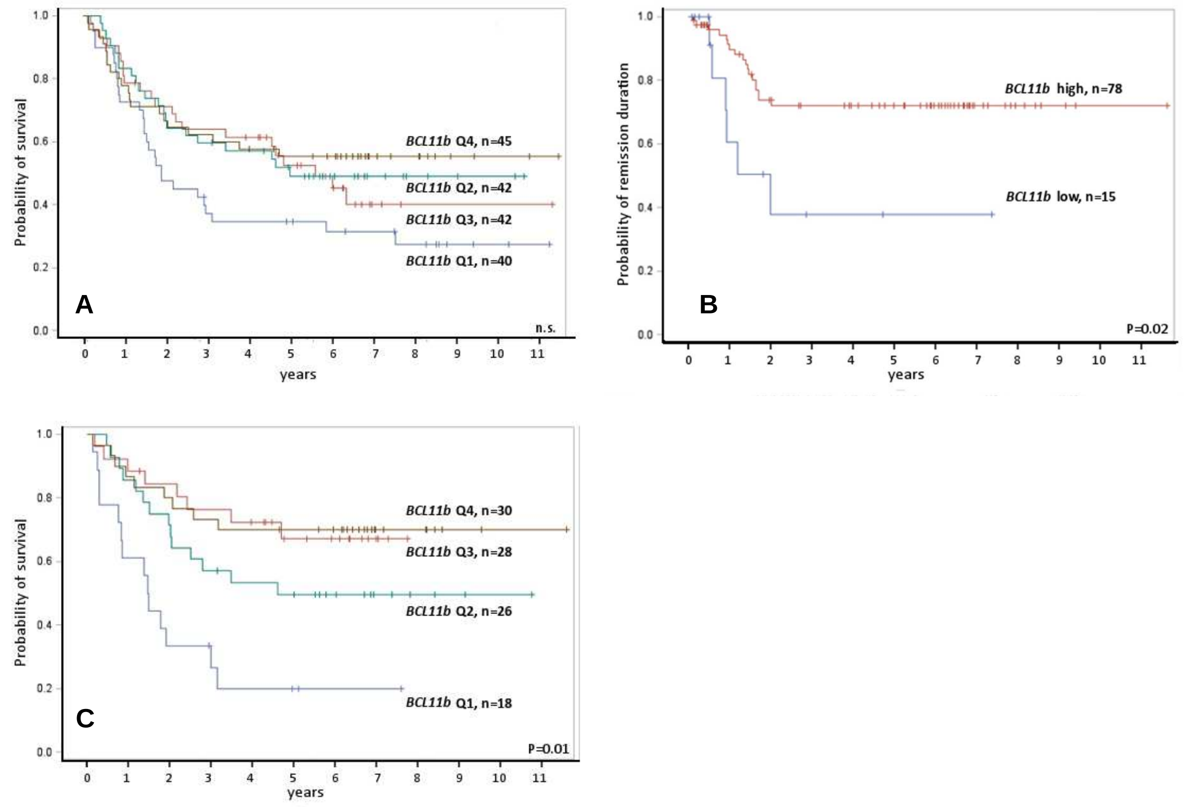


**Figure S2: Kaplan-Meier analyses of overall survival (OS) in T-ALL with respect to *BCL11b* mRNA expression**. **A**: OS of the overall T-ALL cohort according to *BCL11b* expression quartiles (Q1: n=40; 35% OS at 5 yrs; Q2: n=42; 52% OS at 5 yrs; Q3: n=42; 52% OS at 5 yrs; Q4: n=45; 55% OS at 5 yrs; n.s.) **B:** Thymic T-ALL patients with low *BCL11b* expression showed a significant shorter remission duration than thymic patients with high *BCL11b* expression (Q1: n=15, 38% CR at 5 yrs; Q2-4: n=78, 72% CR at 5 yrs; P=0.02). **C**: OS of thymic T-ALL patients according to *BCL11b* expression quartiles (Q1: n=18, 20% at 5 yrs; Q2 n=26, 67% at 5 yrs¸ Q3 n=18, 50% at 5 yrs; Q4 n=30, 70% at 5 yrs; P=0.01).

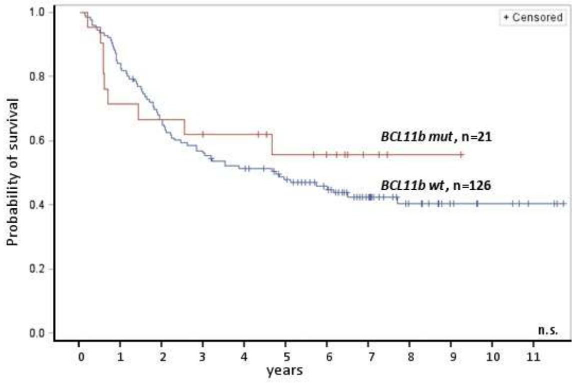


**Figure S3: Kaplan-Meier analysis of overall survival (OS) with respect to the *BCL11b* mutation status.** No significant difference in OS was observed in patients with mutated vs. wild type *BCL11b* (*BCL11b-wt*: n=126; 48% OS at 5 yrs; *BCL11b*-mut: n=21; 56% OS at 5 yrs; n.s.; log-rank test).

| Table S1: Primer sets designed for human *BCL11b* exon 4 (NP_612808.1) | | |
|---|---|---|
| | | |
| **Name** | **Region** | **Sequence** |
| forward1 | exon4_1 | ACGCTGTCTCTGCCTTTCTG |
| reverse1 | exon4_1 | CTCCGAGTCCATGCTGAAG |
| forward2 | exon4_2 | GCTGCTACTGGAGAACGAGAG |
| reverse2 [1] | exon4_2 | CTCGGGTTTCCATAGGACTTC |
| forward3 | exon4_2 | CCACCACGAGAGCGACC |
| reverse3 | exon4_2 | TTGACGTCGTTAGTCAGCAAG |
| forward4 | exon4_2 | GCCAAGCGCATCAAGGTG |

| Table S2: Probe sets two-fold up-regulated in the *BCL11b*-low group compared to *BCL11b*-high group of T-ALL patients (n=86); 229 probe sets, 181 genes | | | |
|---|---|---|---|
| | | | |
| **Probe Set** | **Ratio** | **P-value** | **Gene name** |
| 202625_at | 3.70 | 1.43E-09 | v-yes-1 Yamaguchi sarcoma viral related oncogene homolog |
| 202626_s_at | 3.51 | 2.58E-09 | v-yes-1 Yamaguchi sarcoma viral related oncogene homolog |
| 225659_at | 2.61 | 2.01E-08 | speckle-type POZ protein-like |
| 227013_at | 6.10 | 5.65E-08 | LATS, large tumor suppressor, homolog 2 (Drosophila) |
| 238669_at | 2.36 | 1.73E-07 | prostaglandin-endoperoxide synthase 1 (prostaglandin G/H synthase and cyclooxygenase) |
| 215706_x_at | 2.36 | 2.95E-07 | zyxin |
| 204689_at | 3.08 | 4.66E-07 | hematopoietically expressed homeobox |
| 208890_s_at | 3.22 | 4.97E-07 | plexin B2 |
| 203749_s_at | 2.52 | 5.30E-07 | retinoic acid receptor, alpha |
| 217478_s_at | 3.92 | 8.26E-07 | major histocompatibility complex, class II, DM alpha |
| 208767_s_at | 2.54 | 8.79E-07 | lysosomal protein transmembrane 4 beta |
| 227195_at | 10.12 | 9.36E-07 | zinc finger protein 503 |
| 228188_at | 8.53 | 1.35E-06 | FOS-like antigen 2 |
| 235252_at | 2.38 | 1.72E-06 | kinase suppressor of ras 1 |
| 31845_at | 2.59 | 1.83E-06 | E74-like factor 4 (ets domain transcription factor) |
| 225658_at | 2.59 | 1.83E-06 | speckle-type POZ protein-like |
| 201566_x_at | 2.56 | 1.83E-06 | inhibitor of DNA binding 2, dominant negative helix-loop-helix protein |
| 211302_s_at | 3.73 | 1.94E-06 | phosphodiesterase 4B, cAMP-specific (phosphodiesterase E4 dunce homolog, Drosophila) |
| 203708_at | 3.85 | 2.32E-06 | phosphodiesterase 4B, cAMP-specific (phosphodiesterase E4 dunce homolog, Drosophila) |
| 225955_at | 2.41 | 2.46E-06 | meteorin, glial cell differentiation regulator-like; similar to meteorin, glial cell differentiation regulator-like |
| 212658_at | 2.32 | 2.77E-06 | lipoma HMGIC fusion partner-like 2 |
| 228170_at | 7.81 | 2.94E-06 | oligodendrocyte transcription factor 1 |
| 204490_s_at | 2.40 | 3.49E-06 | CD44 molecule (Indian blood group) |
| 204908_s_at | 2.36 | 4.15E-06 | B-cell CLL/lymphoma 3 |
| 209307_at | 4.04 | 4.39E-06 | SWAP switching B-cell complex 70kDa subunit |
| 225262_at | 3.11 | 4.65E-06 | FOS-like antigen 2 |
| 201163_s_at | 3.18 | 6.89E-06 | insulin-like growth factor binding protein 7 |
| 228964_at | 2.10 | 9.06E-06 | PR domain containing 1, with ZNF domain |
| 209619_at | 4.13 | 1.25E-05 | CD74 molecule, major histocompatibility complex, class II invariant chain |
| 210754_s_at | 2.85 | 1.39E-05 | v-yes-1 Yamaguchi sarcoma viral related oncogene homolog |
| 201536_at | 2.23 | 1.72E-05 | dual specificity phosphatase 3 |
| 204204_at | 2.78 | 1.91E-05 | solute carrier family 31 (copper transporters), member 2 |
| 201162_at | 3.63 | 2.01E-05 | insulin-like growth factor binding protein 7 |
| 211991_s_at | 5.09 | 2.23E-05 | major histocompatibility complex, class II, DP alpha 1 |
| 226397_s_at | 3.67 | 2.35E-05 | - |
| 228831_s_at | 3.03 | 2.47E-05 | guanine nucleotide binding protein (G protein), gamma 7 |
| 212975_at | 2.32 | 2.73E-05 | DENN/MADD domain containing 3 |
| 226459_at | 3.07 | 3.18E-05 | phosphoinositide-3-kinase adaptor protein 1 |
| 203820_s_at | 2.03 | 3.34E-05 | insulin-like growth factor 2 mRNA binding protein 3 |
| 204198_s_at | 2.12 | 3.70E-05 | runt-related transcription factor 3 |
| 218627_at | 2.16 | 3.88E-05 | DNA-damage regulated autophagy modulator 1 |
| 211990_at | 3.51 | 4.29E-05 | major histocompatibility complex, class II, DP alpha 1 |
| 214953_s_at | 2.10 | 4.73E-05 | amyloid beta (A4) precursor protein |
| 204440_at | 2.45 | 4.96E-05 | CD83 molecule |

| Continuation I of Table S2 | | | |
|---|---|---|---|
| **Probe Set** | **Ratio** | **P-value** | **Gene name** |
| 211654_x_at | 3.07 | 5.21E-05 | major histocompatibility complex, class II, DQ beta 1; similar to major histocompatibility complex, class II, DQ beta 1 |
| 201137_s_at | 4.85 | 5.47E-05 | major histocompatibility complex, class II, DP beta 1 |
| 200808_s_at | 2.08 | 5.47E-05 | zyxin |
| 211597_s_at | 3.46 | 5.74E-05 | HOP homeobox |
| 201170_s_at | 2.70 | 5.74E-05 | basic helix-loop-helix family, member e40 |
| 209312_x_at | 2.62 | 6.03E-05 | major histocompatibility complex, class II, DR beta 4; major histocompatibility complex, class II, DR beta 1 |
| 208092_s_at | 2.63 | 6.32E-05 | family with sequence similarity 49, member A |
| 213537_at | 4.64 | 6.63E-05 | major histocompatibility complex, class II, DP alpha 1 |
| 215933_s_at | 2.89 | 6.63E-05 | hematopoietically expressed homeobox |
| 200602_at | 2.84 | 6.63E-05 | amyloid beta (A4) precursor protein |
| 212120_at | 2.64 | 8.40E-05 | ras homolog gene family, member Q; similar to small GTP binding protein TC10 |
| 227425_at | 2.62 | 9.23E-05 | RALBP1 associated Eps domain containing 2 |
| 222582_at | 2.22 | 9.67E-05 | protein kinase, AMP-activated, gamma 2 non-catalytic subunit |
| 230917_at | 2.65 | 1.01E-04 | RNA, 7SK small nuclear |
| 204670_x_at | 3.11 | 1.11E-04 | major histocompatibility complex, class II, DR beta 4; major histocompatibility complex, class II, DR beta 1 |
| 209545_s_at | 2.91 | 1.11E-04 | receptor-interacting serine-threonine kinase 2 |
| 231779_at | 3.11 | 1.16E-04 | interleukin-1 receptor-associated kinase 2 |
| 215193_x_at | 3.00 | 1.16E-04 | major histocompatibility complex, class II, DR beta 3 |
| 224681_at | 2.31 | 1.16E-04 | guanine nucleotide binding protein (G protein) alpha 12 |
| 225344_at | 2.80 | 1.27E-04 | nuclear receptor coactivator 7 |
| 224800_at | 2.25 | 1.27E-04 | WD repeat and FYVE domain containing 1 |
| 214651_s_at | 2.83 | 1.39E-04 | homeobox A9 |
| 206028_s_at | 2.26 | 1.39E-04 | c-mer proto-oncogene tyrosine kinase |
| 214039_s_at | 4.06 | 1.53E-04 | lysosomal protein transmembrane 4 beta |
| 226438_at | 2.35 | 1.53E-04 | syntrophin, beta 1 (dystrophin-associated protein A1, 59kDa, basic component 1) |
| 210136_at | 2.11 | 1.53E-04 | myelin basic protein |
| 218880_at | 2.47 | 1.60E-04 | FOS-like antigen 2 |
| 203603_s_at | 2.35 | 1.67E-04 | zinc finger E-box binding homeobox 2 |
| 208690_s_at | 3.35 | 1.75E-04 | PDZ and LIM domain 1 |
| 203332_s_at | 2.02 | 1.82E-04 | inositol polyphosphate-5-phosphatase, 145kDa |
| 209239_at | 2.51 | 1.99E-04 | nuclear factor of kappa light polypeptide gene enhancer in B-cells 1 |
| 209835_x_at | 2.38 | 2.18E-04 | CD44 molecule (Indian blood group) |
| 212014_x_at | 2.29 | 2.27E-04 | CD44 molecule (Indian blood group) |
| 212611_at | 2.29 | 2.27E-04 | deltex homolog 4 (Drosophila) |
| 210982_s_at | 3.27 | 2.59E-04 | major histocompatibility complex, class II, DR alpha |
| 228461_at | 2.01 | 2.59E-04 | SH3 domain containing ring finger 3 |
| 213348_at | 3.39 | 2.70E-04 | cyclin-dependent kinase inhibitor 1C (p57, Kip2) |
| 209200_at | 2.69 | 2.70E-04 | myocyte enhancer factor 2C |
| 227961_at | 2.66 | 2.82E-04 | cathepsin B |
| 235072_s_at | 3.09 | 2.94E-04 | - |
| 223380_s_at | 2.77 | 3.07E-04 | LATS, large tumor suppressor, homolog 2 (Drosophila) |
| 204249_s_at | 2.33 | 3.07E-04 | LIM domain only 2 (rhombotin-like 1) |
| 232231_at | 2.23 | 3.07E-04 | runt-related transcription factor 2 |
| 209606_at | 2.34 | 3.34E-04 | cytohesin 1 interacting protein |
| 203320_at | 2.13 | 3.34E-04 | SH2B adaptor protein 3 |
| 222780_s_at | 4.54 | 3.64E-04 | brain and acute leukemia, cytoplasmic |
| 204233_s_at | 2.34 | 3.79E-04 | choline kinase alpha |
| 209199_s_at | 3.03 | 4.12E-04 | myocyte enhancer factor 2C |
| 226550_at | 2.64 | 4.12E-04 | - |
| 228181_at | 2.02 | 4.48E-04 | solute carrier family 30 (zinc transporter), member 1 |

| Continuation II of Table S2 | | | |
|---|---|---|---|
| **Probe Set** | **Ratio** | **P-value** | **Gene name** |
| 228153_at | 4.46 | 4.67E-04 | ring finger protein 144B |
| 224909_s_at | 2.28 | 4.67E-04 | phosphatidylinositol-3,4,5-trisphosphate-dependent Rac exchange factor 1 |
| 229221_at | 2.12 | F204670_x_at | CD44 molecule (Indian blood group) |
| 230348_at | 2.76 | 4.86E-04 | LATS, large tumor suppressor, homolog 2 (Drosophila) |
| 207761_s_at | 2.50 | 5.07E-04 | methyltransferase like 7A |
| 202284_s_at | 2.43 | 5.72E-04 | cyclin-dependent kinase inhibitor 1A (p21, Cip1) |
| 229114_at | 2.22 | 5.72E-04 | GRB2-associated binding protein 1 |
| 225524_at | 2.50 | 5.96E-04 | anthrax toxin receptor 2 |
| 208078_s_at | 2.59 | 6.20E-04 | salt-inducible kinase 1 |
| 216944_s_at | 2.47 | 6.45E-04 | inositol 1,4,5-triphosphate receptor, type 1 |
| 224391_s_at | 2.06 | 6.45E-04 | sialic acid acetylesterase |
| 218346_s_at | 2.25 | 6.72E-04 | sestrin 1 |
| 218520_at | 2.03 | 6.99E-04 | TANK-binding kinase 1 |
| 204912_at | 2.17 | 7.87E-04 | interleukin 10 receptor, alpha |
| 209191_at | 4.19 | 8.85E-04 | tubulin, beta 6 |
| 209457_at | 2.52 | 9.94E-04 | dual specificity phosphatase 5 |
| 200839_s_at | 2.31 | 9.94E-04 | cathepsin B |
| 58780_s_at | 2.11 | 9.94E-04 | hypothetical protein FLJ10357 |
| 212907_at | 2.46 | 1.03E-03 | solute carrier family 30 (zinc transporter), member 1 |
| 225567_at | 2.34 | 1.07E-03 | |
| 210665_at | 2.99 | 1.16E-03 | tissue factor pathway inhibitor (lipoprotein-associated coagulation inhibitor) |
| 203932_at | 2.35 | 1.20E-03 | major histocompatibility complex, class II, DM beta |
| 222603_at | 2.54 | 1.25E-03 | endoplasmic reticulum metallopeptidase 1 |
| 206674_at | 2.34 | 1.35E-03 | fms-related tyrosine kinase 3 |
| 239412_at | 2.41 | 1.37E-03 | interferon regulatory factor 5 |
| 209374_s_at | 3.80 | 1.40E-03 | immunoglobulin heavy constant gamma 1 (G1m marker); immunoglobulin heavy constant mu; immunoglobulin heavy variable 3-7; immunoglobulin heavy constant gamma 3 (G3m marker); immunoglobulin heavy variable 3-11 (gene/pseudogene); immunoglobulin heavy variabl |
| 224571_at | 2.01 | 1.40E-03 | interferon regulatory factor 2 binding protein 2 |
| 36711_at | 2.62 | 1.50E-03 | v-maf musculoaponeurotic fibrosarcoma oncogene homolog F (avian) |
| 206478_at | 2.55 | 1.50E-03 | KIAA0125 |
| 208894_at | 3.12 | 1.68E-03 | major histocompatibility complex, class II, DR alpha |
| 208306_x_at | 2.76 | 1.74E-03 | major histocompatibility complex, class II, DR beta 4; major histocompatibility complex, class II, DR beta 1 |
| 235670_at | 2.25 | 1.74E-03 | syntaxin 11 |
| 200953_s_at | 2.41 | 1.87E-03 | cyclin D2 |
| 215087_at | 2.21 | 1.87E-03 | chromosome 15 open reading frame 39 |
| 204882_at | 2.14 | 2.01E-03 | Rho GTPase activating protein 25 |
| 242931_at | 2.71 | 2.09E-03 | LON peptidase N-terminal domain and ring finger 3 |
| 228532_at | 2.26 | 2.24E-03 | chromosome 1 open reading frame 162 |
| 213844_at | 2.94 | 2.68E-03 | homeobox A5 |
| 204491_at | 2.03 | 2.68E-03 | phosphodiesterase 4D, cAMP-specific (phosphodiesterase E3 dunce homolog, Drosophila) |
| 230866_at | 2.03 | 2.68E-03 | cysteinyl leukotriene receptor 1 |
| 238376_at | 2.37 | 2.77E-03 | C-type lectin domain family 16, member A |
| 203037_s_at | 2.12 | 2.77E-03 | metastasis suppressor 1 |
| 203710_at | 2.05 | 2.77E-03 | inositol 1,4,5-triphosphate receptor, type 1 |
| 226657_at | 2.03 | 2.77E-03 | chromosome 17 open reading frame 103 |
| 221773_at | 2.45 | 2.87E-03 | ELK3, ETS-domain protein (SRF accessory protein 2) |
| 212632_at | 2.01 | 2.97E-03 | syntaxin 7 |
| 221766_s_at | 2.14 | 3.07E-03 | family with sequence similarity 46, member A |
| 210835_s_at | 2.18 | 3.18E-03 | C-terminal binding protein 2 |
| 212442_s_at | 2.20 | 3.29E-03 | LAG1 homolog, ceramide synthase 6 |

| Continuation III of Table S2 | | | |
|---|---|---|---|
| **Probe Set** | **Ratio** | **P-value** | **Gene name** |
| 200742_s_at | 2.11 | 3.29E-03 | tripeptidyl peptidase I |
| 225612_s_at | 3.54 | 3.41E-03 | UDP-GlcNAc:betaGal beta-1,3-N-acetylglucosaminyltransferase 5 |
| 203799_at | 2.05 | 3.41E-03 | CD302 molecule; lymphocyte antigen 75 |
| 241756_at | 2.08 | 3.53E-03 | SWI/SNF related, matrix associated, actin dependent regulator of chromatin, subfamily a, member 2 |
| 212086_x_at | 2.03 | 3.65E-03 | lamin A/C |
| 226267_at | 2.42 | 3.77E-03 | Jun dimerization protein 2 |
| 203504_s_at | 2.11 | 3.77E-03 | ATP-binding cassette, sub-family A (ABC1), member 1 |
| 223343_at | 3.20 | 4.46E-03 | membrane-spanning 4-domains, subfamily A, member 7 |
| 227345_at | 2.46 | 4.46E-03 | tumor necrosis factor receptor superfamily, member 10d, decoy with truncated death domain |
| 213258_at | 4.11 | 4.61E-03 | tissue factor pathway inhibitor (lipoprotein-associated coagulation inhibitor) |
| 223501_at | 2.40 | 4.76E-03 | tumor necrosis factor (ligand) superfamily, member 13b |
| 225589_at | 2.27 | 4.76E-03 | SH3 domain containing ring finger 1 |
| 210664_s_at | 2.67 | 4.92E-03 | tissue factor pathway inhibitor (lipoprotein-associated coagulation inhibitor) |
| 202724_s_at | 2.25 | 4.92E-03 | forkhead box O1 |
| 212827_at | 3.87 | 5.25E-03 | immunoglobulin heavy constant gamma 1 (G1m marker); immunoglobulin heavy constant mu; immunoglobulin heavy variable 3-7; immunoglobulin heavy constant gamma 3 (G3m marker); immunoglobulin heavy variable 3-11 (gene/pseudogene); immunoglobulin heavy variabl |
| 205027_s_at | 2.58 | 5.25E-03 | mitogen-activated protein kinase kinase kinase 8 |
| 227200_at | 2.09 | 5.43E-03 | |
| 212509_s_at | 2.19 | 5.79E-03 | matrix-remodelling associated 7 |
| 230233_at | 2.27 | 5.98E-03 | RasGEF domain family, member 1B |
| 238509_at | 2.23 | 6.17E-03 | cullin 1 |
| 227020_at | 2.10 | 6.17E-03 | yippee-like 2 (Drosophila) |
| 217707_x_at | 2.04 | 6.58E-03 | SWI/SNF related, matrix associated, actin dependent regulator of chromatin, subfamily a, member 2 |
| 229560_at | 2.23 | 6.79E-03 | toll-like receptor 8 |
| 225372_at | 2.15 | 7.23E-03 | chromosome 10 open reading frame 54 |
| 206310_at | 2.02 | 7.69E-03 | serine peptidase inhibitor, Kazal type 2 (acrosin-trypsin inhibitor) |
| 206631_at | 2.09 | 7.94E-03 | prostaglandin E receptor 2 (subtype EP2), 53kDa |
| 209306_s_at | 4.23 | 8.19E-03 | SWAP switching B-cell complex 70kDa subunit |
| 242064_at | 2.03 | 8.19E-03 | sidekick homolog 2 (chicken) |
| 209676_at | 2.76 | 8.44E-03 | tissue factor pathway inhibitor (lipoprotein-associated coagulation inhibitor) |
| 209543_s_at | 2.48 | 8.71E-03 | CD34 molecule |
| 228314_at | 2.21 | 9.25E-03 | leucine rich repeat containing 8 family, member C |
| 225033_at | 2.19 | 9.25E-03 | ST3 beta-galactoside alpha-2,3-sialyltransferase 1 |
| 220603_s_at | 2.24 | 9.83E-03 | multiple C2 domains, transmembrane 2 |
| 242794_at | 2.36 | 1.04E-02 | mastermind-like 3 (Drosophila) |
| 227983_at | 2.41 | 1.08E-02 | Rab interacting lysosomal protein-like 2 |
| 205609_at | 3.27 | 1.14E-02 | angiopoietin 1 |
| 200696_s_at | 2.10 | 1.14E-02 | gelsolin (amyloidosis, Finnish type) |
| 226452_at | 2.26 | 1.18E-02 | pyruvate dehydrogenase kinase, isozyme 1 |
| 226844_at | 2.11 | 1.21E-02 | MOB1, Mps One Binder kinase activator-like 2B (yeast) |
| 225337_at | 2.11 | 1.21E-02 | abhydrolase domain containing 2 |
| 239598_s_at | 2.01 | 1.25E-02 | lysophosphatidylcholine acyltransferase 2 |
| 212446_s_at | 2.53 | 1.28E-02 | LAG1 homolog, ceramide synthase 6 |
| 235463_s_at | 2.13 | 1.28E-02 | LAG1 homolog, ceramide synthase 6 |
| 229450_at | 2.73 | 1.36E-02 | interferon-induced protein with tetratricopeptide repeats 3 |
| 238732_at | 2.25 | 1.36E-02 | collagen, type XXIV, alpha 1 |
| 216268_s_at | 2.21 | 1.44E-02 | jagged 1 (Alagille syndrome) |
| 218858_at | 2.06 | 1.53E-02 | DEP domain containing 6 |
| 213902_at | 2.14 | 1.57E-02 | N-acylsphingosine amidohydrolase (acid ceramidase) 1 |

| Continuation IV of Table S2 | | | |
|---|---|---|---|
| **Probe Set** | **Ratio** | **P-value** | **Gene name** |
| 201204_s_at | 2.19 | 1.62E-02 | ribosome binding protein 1 homolog 180kDa (dog) |
| 201200_at | 2.14 | 1.62E-02 | cellular repressor of E1A-stimulated genes 1 |
| 231972_at | 2.04 | 1.62E-02 | |
| 226818_at | 3.20 | 1.66E-02 | macrophage expressed 1 |
| 206544_x_at | 2.52 | 1.66E-02 | SWI/SNF related, matrix associated, actin dependent regulator of chromatin, subfamily a, member 2 |
| 211612_s_at | 2.41 | 1.66E-02 | interleukin 13 receptor, alpha 1 |
| 225673_at | 2.07 | 1.66E-02 | myeloid-associated differentiation marker |
| 221523_s_at | 2.14 | 1.76E-02 | Ras-related GTP binding D |
| 202768_at | 2.28 | 1.86E-02 | FBJ murine osteosarcoma viral oncogene homolog B |
| 205098_at | 2.42 | 2.08E-02 | chemokine (C-C motif) receptor 1 |
| 214696_at | 2.11 | 2.13E-02 | chromosome 17 open reading frame 91 |
| 210797_s_at | 2.18 | 2.44E-02 | 2'-5'-oligoadenylate synthetase-like |
| 204203_at | 2.02 | 2.44E-02 | CCAAT/enhancer binding protein (C/EBP), gamma |
| 203753_at | 2.44 | 2.51E-02 | transcription factor 4 |
| 215285_s_at | 2.34 | 2.51E-02 | putative homeodomain transcription factor 1 |
| 209774_x_at | 2.46 | 2.57E-02 | chemokine (C-X-C motif) ligand 2 |
| 201218_at | 2.41 | 2.57E-02 | C-terminal binding protein 2 |
| 224973_at | 2.23 | 2.64E-02 | family with sequence similarity 46, member A |
| 202429_s_at | 2.14 | 2.64E-02 | protein phosphatase 3 (formerly 2B), catalytic subunit, alpha isoform |
| 210999_s_at | 2.98 | 2.71E-02 | growth factor receptor-bound protein 10 |
| 228573_at | 2.09 | 2.86E-02 | anthrax toxin receptor 2 |
| 238002_at | 3.86 | 2.93E-02 | golgi integral membrane protein 4 |
| 212314_at | 2.47 | 2.93E-02 | KIAA0746 protein |
| 222698_s_at | 2.41 | 2.93E-02 | Impact homolog (mouse) |
| 239835_at | 2.29 | 2.93E-02 | kelch repeat and BTB (POZ) domain containing 8 |
| 218284_at | 2.61 | 3.09E-02 | SMAD family member 3 |
| 204614_at | 2.12 | 3.51E-02 | serpin peptidase inhibitor, clade B (ovalbumin), member 2 |
| 200838_at | 2.44 | 3.60E-02 | cathepsin B |
| 205401_at | 2.51 | 3.88E-02 | alkylglycerone phosphate synthase |
| 209301_at | 2.17 | 4.07E-02 | carbonic anhydrase II |
| 212719_at | 2.14 | 4.07E-02 | PH domain and leucine rich repeat protein phosphatase 1 |
| 212592_at | 3.13 | 4.28E-02 | immunoglobulin J polypeptide, linker protein for immunoglobulin alpha and mu polypeptides |
| 221491_x_at | 2.76 | 4.28E-02 | major histocompatibility complex, class II, DR beta 3 |
| 217997_at | 2.21 | 4.38E-02 | pleckstrin homology-like domain, family A, member 1 |
| 201887_at | 4.13 | 4.49E-02 | interleukin 13 receptor, alpha 1 |
| 205239_at | 2.44 | 4.49E-02 | amphiregulin; amphiregulin B |
| 223502_s_at | 2.16 | 4.49E-02 | tumor necrosis factor (ligand) superfamily, member 13b |
| 214449_s_at | 2.25 | 4.60E-02 | ras homolog gene family, member Q; similar to small GTP binding protein TC10 |

| **Table S3: Probe sets two-fold up-regulated in the *BCL11b*-high group compared to the *BCL11b*-high group of T-ALL patients (n=86); (200 probe sets, 162 genes)** | | | |
|---|---|---|---|
| **Probe Set** | **Ratio** | **P-value** | **Gene name** |
| 219528_s_at | 7.24 | 9.35E-14 | B-cell CLL/lymphoma 11B (zinc finger protein) |
| 206804_at | 4.06 | 2.95E-10 | CD3g molecule, gamma (CD3-TCR complex) |
| 221558_s_at | 3.59 | 9.95E-08 | lymphoid enhancer-binding factor 1 |
| 202789_at | 2.96 | 1.98E-07 | phospholipase C, gamma 1 |
| 227766_at | 3.45 | 3.60E-07 | ligase IV, DNA, ATP-dependent |
| 213623_at | 3.41 | 4.97E-07 | kinesin family member 3A |
| 207434_s_at | 7.18 | 1.27E-06 | FXYD domain containing ion transport regulator 2 |
| 211005_at | 3.65 | 1.35E-06 | linker for activation of T cells |
| 226864_at | 5.60 | 1.83E-06 | protein kinase (cAMP-dependent, catalytic) inhibitor alpha |
| 213539_at | 2.41 | 2.19E-06 | CD3d molecule, delta (CD3-TCR complex) |
| 204890_s_at | 3.41 | 2.32E-06 | lymphocyte-specific protein tyrosine kinase |
| 204777_s_at | 6.19 | 3.11E-06 | mal, T-cell differentiation protein |
| 205674_x_at | 6.71 | 3.49E-06 | FXYD domain containing ion transport regulator 2 |
| 222803_at | 3.41 | 4.65E-06 | phosphoribosyl transferase domain containing 1 |
| 220429_at | 2.72 | 4.92E-06 | N-deacetylase/N-sulfotransferase (heparan glucosaminyl) 3 |
| 243362_s_at | 5.07 | 5.51E-06 | hypothetical protein LOC641518 |
| 205255_x_at | 2.61 | 7.28E-06 | transcription factor 7 (T-cell specific, HMG-box) |
| 214032_at | 2.33 | 7.69E-06 | zeta-chain (TCR) associated protein kinase 70kDa |
| 222288_at | 4.11 | 9.06E-06 | unc-5 homolog B |
| 205590_at | 4.64 | 9.57E-06 | RAS guanyl releasing protein 1 (calcium and DAG-regulated) |
| 202850_at | 2.13 | 1.01E-05 | ATP-binding cassette, sub-family D (ALD), member 3 |
| 219812_at | 2.94 | 1.25E-05 | poliovirus receptor related immunoglobulin domain containing |
| 235733_at | 2.98 | 1.25E-05 | glucoside xylosyltransferase 2 |
| 36545_s_at | 2.03 | 1.32E-05 | Sfi1 homolog, spindle assembly associated (yeast) |
| 225591_at | 2.13 | 1.81E-05 | F-box protein 25 |
| 224719_s_at | 2.18 | 1.81E-05 | chromosome 12 open reading frame 57 |
| 241871_at | 2.97 | 1.81E-05 | calcium/calmodulin-dependent protein kinase IV |
| 233198_at | 2.14 | 2.01E-05 | golgi autoantigen, golgin subfamily a, 2-like 1 |
| 219532_at | 3.87 | 2.35E-05 | elongation of very long chain fatty acids (FEN1/Elo2, SUR4/Elo3, yeast)-like 4 |
| 216705_s_at | 2.41 | 2.35E-05 | adenosine deaminase |
| 205871_at | 2.09 | 2.60E-05 | plasminogen-like B2; plasminogen-like B1 |
| 227077_at | 2.28 | 2.88E-05 | zinc finger protein 286A |
| 213060_s_at | 2.97 | 3.18E-05 | chitinase 3-like 2 |
| 201014_s_at | 2.08 | 3.34E-05 | phosphoribosylaminoimidazole carboxylase, phosphoribosylaminoimidazole succinocarboxamide synthetase |
| 202534_x_at | 2.40 | 3.52E-05 | dihydrofolate reductase |
| 241074_at | 3.48 | 4.73E-05 | immunoglobulin heavy variable 3-33-2 pseudogene |
| 228792_at | 2.38 | 4.96E-05 | solute carrier family 24, member 5 |
| 220865_s_at | 3.16 | 5.21E-05 | prenyl (decaprenyl) diphosphate synthase, subunit 1 |
| 213556_at | 2.18 | 5.47E-05 | similar to R28379_1 |
| 211535_s_at | 2.62 | 5.47E-05 | fibroblast growth factor receptor 1 |
| 206150_at | 3.19 | 5.47E-05 | CD27 molecule |
| 210094_s_at | 4.47 | 5.74E-05 | par-3 partitioning defective 3 homolog (C. elegans) |
| 232001_at | 2.42 | 6.03E-05 | hypothetical protein LOC439949 |
| 219156_at | 2.17 | 6.32E-05 | synaptojanin 2 binding protein |
| 205456_at | 2.15 | 6.63E-05 | CD3e molecule, epsilon (CD3-TCR complex) |

| Continuation I of Table S3 | | | |
|---|---|---|---|
| **Probe Set** | **Ratio** | **P-value** | **Gene name** |
| 224926_at | 3.36 | 7.65E-05 | exocyst complex component 4 |
| 226925_at | 2.60 | 8.40E-05 | acid phosphatase-like 2 |
| 205673_s_at | 2.11 | 8.81E-05 | ankyrin repeat and SOCS box-containing 9 |
| 244033_at | 4.07 | 9.23E-05 | chromosome 14 open reading frame 145 |
| 241859_at | 3.93 | 9.67E-05 | phospholipase C-like 1 |
| 39248_at | 5.67 | 9.67E-05 | aquaporin 3 (Gill blood group) |
| 229533_x_at | 2.38 | 1.01E-04 | zinc finger protein 680 |
| 229202_at | 2.69 | 1.16E-04 | pecanex-like 2 (Drosophila) |
| 206460_at | 4.07 | 1.22E-04 | adherens junctions associated protein 1 |
| 217147_s_at | 2.45 | 1.27E-04 | T cell receptor associated transmembrane adaptor 1 |
| 235371_at | 2.92 | 1.46E-04 | glycosyltransferase 8 domain containing 4 |
| 213193_x_at | 2.68 | 1.53E-04 | T cell receptor beta variable 19; T cell receptor beta constant 1 |
| 238593_at | 2.41 | 1.53E-04 | chromosome 11 open reading frame 80 |
| 205005_s_at | 2.32 | 1.75E-04 | N-myristoyltransferase 2 |
| 218472_s_at | 2.06 | 1.82E-04 | pelota homolog (Drosophila) |
| 211796_s_at | 2.59 | 2.08E-04 | T cell receptor beta constant 2; T cell receptor beta variable 5-4; T cell receptor beta variable 3-1; T cell receptor beta variable 7-2 |
| 228988_at | 2.47 | 2.37E-04 | zinc finger protein 711 |
| 221606_s_at | 3.48 | 2.37E-04 | nucleosomal binding protein 1 |
| 210487_at | 2.65 | 2.59E-04 | deoxynucleotidyltransferase, terminal |
| 242292_at | 2.21 | 2.70E-04 | chromosome X open reading frame 50B; chromosome X open reading frame 50 |
| 205831_at | 3.19 | 2.82E-04 | CD2 molecule |
| 228455_at | 2.14 | 2.94E-04 | RNA binding motif protein 15 |
| 201518_at | 2.20 | 3.49E-04 | chromobox homolog 1 (HP1 beta homolog Drosophila ) |
| 228280_at | 2.52 | 3.79E-04 | zinc finger CCCH-type, antiviral 1-like |
| 205559_s_at | 2.31 | 4.30E-04 | proprotein convertase subtilisin/kexin type 5 |
| 241864_x_at | 2.98 | 4.30E-04 | - |
| 218984_at | 2.15 | 4.48E-04 | pseudouridylate synthase 7 homolog (S. cerevisiae) |
| 227350_at | 2.49 | 4.67E-04 | helicase, lymphoid-specific |
| 208146_s_at | 2.49 | 4.86E-04 | carboxypeptidase, vitellogenic-like |
| 228049_x_at | 2.30 | 5.07E-04 | small nucleolar RNA, C/D box 60 |
| 201194_at | 2.07 | 5.07E-04 | selenoprotein W, 1 |
| 213958_at | 2.01 | 5.07E-04 | CD6 molecule |
| 206545_at | 4.16 | 5.49E-04 | CD28 molecule |
| 228303_at | 2.23 | 5.72E-04 | UDP-N-acetyl-alpha-D-galactosamine:polypeptide N-acetylgalactosaminyltransferase 6 (GalNAc-T6) |
| 243279_at | 2.15 | 5.72E-04 | - |
| 211052_s_at | 2.40 | 5.96E-04 | tubulin folding cofactor D |
| 229851_s_at | 2.33 | 5.96E-04 | chromosome 11 open reading frame 54 |
| 221521_s_at | 2.09 | 6.20E-04 | GINS complex subunit 2 (Psf2 homolog) |
| 213939_s_at | 2.54 | 6.72E-04 | RUN and FYVE domain containing 3 |
| 235383_at | 2.39 | 6.72E-04 | myosin VIIB |
| 223530_at | 2.00 | 7.56E-04 | tudor and KH domain containing |
| 204849_at | 2.03 | 7.56E-04 | transcription factor-like 5 (basic helix-loop-helix) |
| 201792_at | 2.73 | 7.56E-04 | AE binding protein 1 |
| 218477_at | 2.09 | 8.18E-04 | transmembrane protein 14A |
| 227921_at | 2.35 | 8.85E-04 | ENSG00000212911 |
| 204142_at | 2.50 | 1.03E-03 | enolase superfamily member 1 |
| 227790_at | 2.35 | 1.07E-03 | ubiquitin-conjugating enzyme E2C binding protein |
| 203362_s_at | 2.05 | 1.07E-03 | MAD2 mitotic arrest deficient-like 1 (yeast) |
| 236846_at | 2.29 | 1.07E-03 | hypothetical protein LOC284757 |
| 48808_at | 2.46 | 1.11E-03 | dihydrofolate reductase |

| Continuation II of Table S3 | | | |
|---|---|---|---|
| **Probe Set** | **Ratio** | **P-value** | **Gene name** |
| 222771_s_at | 2.14 | 1.16E-03 | myelin expression factor 2 |
| 204159_at | 2.45 | 1.20E-03 | cyclin-dependent kinase inhibitor 2C (p18, inhibits CDK4) |
| 203238_s_at | 3.11 | 1.25E-03 | Notch homolog 3 (Drosophila) |
| 236451_at | 2.20 | 1.25E-03 | - |
| 204718_at | 2.68 | 1.40E-03 | EPH receptor B6 |
| 211709_s_at | 2.13 | 1.40E-03 | C-type lectin domain family 11, member A |
| 213385_at | 2.33 | 1.45E-03 | chimerin (chimaerin) 2 |
| 225386_s_at | 2.47 | 1.50E-03 | heterogeneous nuclear ribonucleoprotein L-like |
| 219109_at | 2.05 | 1.62E-03 | sperm associated antigen 16 |
| 228423_at | 2.06 | 1.74E-03 | microtubule-associated protein 9 |
| 221081_s_at | 2.50 | 1.94E-03 | DENN/MADD domain containing 2D |
| 221286_s_at | 2.15 | 2.01E-03 | hypothetical protein MGC29506 |
| 212672_at | 2.06 | 2.09E-03 | similar to Serine-protein kinase ATM (Ataxia telangiectasia mutated) (A-T, mutated); ataxia telangiectasia mutated |
| 238430_x_at | 2.35 | 2.09E-03 | schlafen family member 5 |
| 205758_at | 3.01 | 2.16E-03 | CD8a molecule |
| 227266_s_at | 2.32 | 2.32E-03 | FYN binding protein (FYB-120/130) |
| 214835_s_at | 2.22 | 2.49E-03 | similar to sucb; succinate-CoA ligase, GDP-forming, beta subunit |
| 243996_at | 2.07 | 2.58E-03 | natriuretic peptide receptor B/guanylate cyclase B (atrionatriuretic peptide receptor B) |
| 212813_at | 2.20 | 2.58E-03 | junctional adhesion molecule 3 |
| 219148_at | 2.31 | 2.68E-03 | PDZ binding kinase |
| 232238_at | 2.54 | 2.68E-03 | asp (abnormal spindle) homolog, microcephaly associated (Drosophila) |
| 204759_at | 3.35 | 2.77E-03 | regulator of chromosome condensation (RCC1) and BTB (POZ) domain containing protein 2 |
| 241505_at | 2.72 | 2.77E-03 | - |
| 242136_x_at | 2.11 | 2.97E-03 | C-terminal binding protein 2 pseudogene |
| 226548_at | 2.06 | 3.07E-03 | SH3-binding domain kinase 1 |
| 63009_at | 2.13 | 3.18E-03 | SHQ1 homolog (S. cerevisiae) |
| 232180_at | 2.09 | 3.53E-03 | UDP-glucose pyrophosphorylase 2 |
| 210676_x_at | 2.36 | 3.53E-03 | RANBP2-like and GRIP domain containing 5; RANBP2-like and GRIP domain containing 4; RANBP2-like and GRIP domain containing 3; RANBP2-like and GRIP domain containing 8; RANBP2-like and GRIP domain containing 7; RANBP2-like and GRIP domain containing 6 |
| 200897_s_at | 2.55 | 3.53E-03 | palladin, cytoskeletal associated protein |
| 212372_at | 2.27 | 3.53E-03 | myosin, heavy chain 10, non-muscle |
| 207445_s_at | 2.51 | 4.03E-03 | chemokine (C-C motif) receptor 9 |
| 232392_at | 2.24 | 4.46E-03 | splicing factor, arginine/serine-rich 3 |
| 203764_at | 2.28 | 4.76E-03 | discs, large (Drosophila) homolog-associated protein 5 |
| 225619_at | 2.06 | 4.92E-03 | SLAIN motif family, member 1 |
| 227552_at | 2.05 | 4.92E-03 | septin 1 |
| 205830_at | 2.13 | 4.92E-03 | calmegin |
| 244043_at | 2.41 | 5.08E-03 | transcription factor Dp-2 (E2F dimerization partner 2) |
| 203394_s_at | 3.35 | 5.08E-03 | hairy and enhancer of split 1, (Drosophila) |
| 205267_at | 2.29 | 5.43E-03 | POU class 2 associating factor 1 |
| 239253_at | 2.15 | 5.43E-03 | - |
| 225655_at | 2.08 | 5.60E-03 | ubiquitin-like with PHD and ring finger domains 1 |
| 209569_x_at | 2.03 | 5.79E-03 | DNA segment on chromosome 4 (unique) 234 expressed sequence |
| 209754_s_at | 2.00 | 6.58E-03 | thymopoietin |
| 226610_at | 2.27 | 6.79E-03 | centromere protein V |
| 243016_at | 2.36 | 7.01E-03 | thymidylate synthetase |
| 226365_at | 2.07 | 8.19E-03 | - |
| 202431_s_at | 2.11 | 8.71E-03 | v-myc myelocytomatosis viral oncogene homolog (avian) |
| 240458_at | 2.42 | 8.71E-03 | inositol 1,4,5-triphosphate receptor, type 2 |

| Continuation III of Table S3 | | | |
|---|---|---|---|
| **Probe Set** | **Ratio** | **P-value** | **Gene name** |
| 222344_at | 3.04 | 8.98E-03 | chromosome 5 open reading frame 13 |
| 218437_s_at | 2.00 | 1.04E-02 | leucine zipper transcription factor-like 1 |
| 226587_at | 2.05 | 1.11E-02 | small nuclear ribonucleoprotein polypeptide N; SNRPN upstream reading frame |
| 227663_at | 2.03 | 1.11E-02 | - |
| 239930_at | 2.10 | 1.21E-02 | UDP-N-acetyl-alpha-D-galactosamine:polypeptide N-acetylgalactosaminyltransferase 2 (GalNAc-T2) |
| 202412_s_at | 2.06 | 1.21E-02 | ubiquitin specific peptidase 1 |
| 226685_at | 2.04 | 1.21E-02 | syntrophin, beta 2 (dystrophin-associated protein A1, 59kDa, basic component 2) |
| 216620_s_at | 2.71 | 1.28E-02 | Rho guanine nucleotide exchange factor (GEF) 10 |
| 213093_at | 2.80 | 1.28E-02 | protein kinase C, alpha |
| 236140_at | 2.04 | 1.28E-02 | glutamate-cysteine ligase, modifier subunit |
| 205449_at | 2.20 | 1.36E-02 | SAC3 domain containing 1 |
| 226085_at | 2.23 | 1.36E-02 | chromobox homolog 5 (HP1 alpha homolog, Drosophila) |
| 203404_at | 2.05 | 1.53E-02 | armadillo repeat containing, X-linked 2 |
| 204825_at | 2.02 | 1.76E-02 | maternal embryonic leucine zipper kinase |
| 209884_s_at | 2.04 | 1.81E-02 | solute carrier family 4, sodium bicarbonate cotransporter, member 7 |
| 204146_at | 2.02 | 1.81E-02 | RAD51 associated protein 1 |
| 224496_s_at | 2.26 | 1.96E-02 | transmembrane protein 107 |
| 230493_at | 2.46 | 2.02E-02 | shisa homolog 2 (Xenopus laevis) |
| 200862_at | 2.19 | 2.08E-02 | 24-dehydrocholesterol reductase |
| 210239_at | 2.38 | 2.38E-02 | iroquois homeobox 5 |
| 235425_at | 2.79 | 2.57E-02 | shugoshin-like 2 (S. pombe) |
| 210407_at | 2.13 | 2.71E-02 | protein phosphatase 1A (formerly 2C), magnesium-dependent, alpha isoform |
| 239946_at | 2.41 | 3.33E-02 | KIAA0922 |
| 236293_at | 2.38 | 3.78E-02 | ras homolog gene family, member H |
| 218974_at | 2.13 | 3.88E-02 | sine oculis binding protein homolog (Drosophila) |
| 243808_at | 2.23 | 3.88E-02 | cyclin-dependent kinase 6 |
| 227884_at | 2.14 | 4.38E-02 | TAF15 RNA polymerase II, TATA box binding protein (TBP)-associated factor, 68kDa |
| 243589_at | 2.43 | 4.49E-02 | KIAA1267 |
| 243031_at | 2.15 | 4.71E-02 | reticulon 4 |
| 210387_at | 3.22 | 4.94E-02 | histone cluster 1, H2bi; histone cluster 1, H2bg; histone cluster 1, H2be; histone cluster 1, H2bf; histone cluster 1, H2bc |

| **Table S4: Molecular characteristics of T-ALL patients with respect to *BCL11b* expression** | | | | |
|---|---|---|---|---|
| **Characteristics** | | ***BCL11b*-low** **Q1** | ***BCL11b*-high** **Q2-4** | **P-value** |
| **n** | | 49 | 146 | |
| **Gene mRNA expression levels** | | | | |
| *IGFBP7* | n | 48 | 141 | 0.02 |
| | Median | 1.4 | 0.5 | |
| | Range | 0-45.3 | 0-79.1 | |
| *BAALC* | n | 34 | 130 | n.s. |
| | Median | 0.1 | 0.1 | |
| | Range | 0-7.2 | 0-160.3 | |
| *ERG* | n | 45 | 124 | n.s. |
| | Median | 6.1 | 7.3 | |
| | Range | 0-62.7 | 0-136.7 | |
| *WT1* | n | 49 | 135 | n.s. |
| | Median | 0 | 0 | |
| | Range | 0-0.4 | 0-1.5 | |
| *GATA3* | n | 28 | 74 | <0.01 |
| | Median | 2.1 | 5.7 | |
| | Range | 0.1-15.6 | 0.2-42.7 | |
| **Mutations** | | | | |
| *WT1* mut/total (%) | | 8/49 (16.3%) | 16/146 (11%) | n.s. |
| *NOTCH1* mut/total (%) | | 10/18 (55.6%) | 43/67 (64.1%) | n.s. |
| **TCR rearrangement** | | | | |
| clonal/total (%) | | 16/32 (50) | 44/55 (80) | 0.01 |

| **Table S5: Molecular characteristics of thymic T-ALL patients with respect to *BCL11b* expression** | | | |
|---|---|---|---|
| | | | |
| **Characteristics** | ***BCL11b*-low Q1** | ***BCL11b*-high Q2-4** | **P-value** |
| **T-ALL** | | | |
| **n** | 18 | 94 | |
| | | | |
| **Gene mRNA expression levels** | | | |
| *BAALC* | | | n.s. |
| Median | 0 | 0.1 | |
| Range | 0.0-0.3 | 0.0-10.4 | |
| *ERG* | | | n.s. |
| Median | 1.1 | 1.2 | |
| Range | 0.2-7.8 | 0.1-6.6 | |
| *IGFBP7* | | | n.s. |
| Median | 0.3 | 0.4 | |
| Range | 0.0-16.2 | 0.0-12.5 | |
| *WT1* | | | n.s. |
| Median | 0 | 0 | |
| Range | 0.0-0.1 | 0.0-0.6 | |
| *GATA3* | | | <0.01 |
| Median | 1.6 | 5.1 | |
| Range | 0.3-5.7 | 1.2-25.3 | |
| **Mutations** | | | |
| *WT1* no (%) | 3/18 (17%) | 10/94 (11%) | n.s. |
| *NOTCH1* no (%) | 4/5 (80%) | 30/40 (75%) | n.s. |
| **TCR rearrangement** | | | n.s. |
| clonal/total (%) | 7/13 (54%) | 23/38 (61%) | |

| **Table S6: *BCL11b* exon 4 mutations in T-ALL**<br>*BCL11b*, transcript NM_138576.2 | | | |
|---|---|---|---|
| **T-ALL ID** | **Base change** | **AS change** | **Location** |
| 3 | C792T | L470F | ZF3 |
| 3 | C844T | P454L | ZF2 |
| 4 | G851A | R472A | ZF3 |
| 5 | 1128 ins(TT) | E590fs | fs |
| 29 | C780T | T450M | ZF2 |
| 30 | C844T | L470F | ZF3 |
| 31 | 1705 ds(16bp) | P783fs | fs |
| 31 | C797A | Y455* | stop, ZF3 |
| 34 | 355ds | F317fs | fs |
| 38 | G851A | R472A | ZF3 |
| 40 | C780T | T450M | ZF2 |
| 41 | G320T | G306C | ZF1 |
| 50 | 498ds | S358fs | fs |
| 53 | 90ds | Q238fs | ZF1 |
| 57 | G718T | E430* | stop, ZF1 |
| 76 | 1109 ins(GG) | A584fs | fs |
| 87 | C865T | T476M | ZF3 |
| 99 | C850T | R472C | ZF3 |
| 101 | G812C | C460S | ZF3 |
| 105 | C780T | T450M | ZF2 |
| 108 | C499A | S358* | stop |
| 110 | A1903G | Q848R | - |
| 111 | 611ds | F395fs | fs |
| 120 | 817ds | D461fs | ZF3 |
| 139 | 828ds | S465fs | ZF3 |

Abbreviations: ds: deletion, ins: insertion, fs: frame shift, bp: base pair,
*: stop of translation, ZF= zinc finger

| **Table S7: Clinical characteristics of GMALL T-ALL patients with respect to *BCL11b* mutations** | | | |
|---|---|---|---|
| | | | |
| **Characteristics** | ***BCL11b wt*** | ***BCL11b mut*** | **P-value** |
| **n** | 126 (86%) | 21 (14%) | n.s. |
| | | | |
| ***BCL11b* Expression** | | | n.s. |
| Median | 0.5 | 0.8 | |
| Range | 0.0-12.3 | 0.0-2.7 | |
| **Age** | | | n.s. |
| 15-35 yrs | 72 | 13 | |
| 36-55 yrs | 47 | 7 | |
| 56-65 yrs | 7 | 1 | |
| **Sex** | | | n.s. |
| Female % | 21% | 29% | |
| **WBC, x $10^9$/l** | | | n.s. |
| <30,000 | 52 | 6 | |
| 30-100,000 | 44 | 10 | |
| >100,000 | 25 | 5 | |
| **T-ALL subtyp** | | | 0.03 |
| Early n (%) | 33 (26%) | 1 (5%) | |
| Mature n (%) | 23 (18%) | 2 (9.5%) | |
| Thymic n (%) | 70 (56%) | 18 (85.5%) | |
| **Response to induction therapy*** | | | |
| CR n (%) | 116 (96%) | 18 (86%) | n.s. |
| ED n (%) | 2 (2%) | 1 (5%) | n.s. |
| Failure n (%) | 3 (2%) | 2 (10%) | n.s. |

Abbreviations: CR = complete remission, ED = early death
*evaluable = in 142 patients information of response to induction therapy was available

**Table S8: Members of the German Multicenter Study Group for Adult ALL**

*Augsburg*: Zentralklinikum (G. Schlimok, D. Oruzio, C. Schmid)
*Berlin*: Charité Campus Benjamin Franklin (E. Thiel, S. Schwartz)
*Berlin*: Vivantes-Klinikum (J. Beyer, M. Hackenthal)
*Berlin*: Charité Campus Virchow (R. Arnold, T. Terwey)
*Berlin*: Charité Campus Buch (W.-D. Ludwig, R. Ratei)
*Berlin*: Vivantes Klinikum Neukölln (M. de Wit, A. Grüneisen, B. Kallinich)
*Berlin*: St.-Hedwig-Krankenhaus (K,-M. Derwahl, H. Englisch, B. Oldenkott)
*Bielefeld*: Franziskus-Hospital (H.J. Weh, A. Zumsprekel)
*Bochum*: Knappschaftskrankenhaus (W. Schmiegel, C. Teschendorf)
*Bremen*: Klinikum Mitte (B. Hertenstein, S. Kaun)
*Bremen*: Evangelische Diakonissenanstalt (K. H. Pflüger, J. Kullmer)
*Bremerhaven*: Ev. Diakonissenanstalt (H.-H. Heidtmann, A. Pott)
*Chemnitz*: Klinikum (M. Haenel, A. Morgner)
*Cottbus*: Carl-Thiem-Klinikum (H.B. Steinhauer, N. Peter, K. Petzold)
*Dessau*: Städt. Klinikum (A. Florschütz, O. Kellner)
*Dortmund*: St. Johannes-Hospital (H. Pielken, H. Hindahl, V. Hagen)
*Dresden*: Klinikum Carl Gustav Carus (G. Ehninger, R. Naumann, M. Schaich)
*Duisburg*: St. Johannes-Hospital (C. Aul, A. Giagounidis)
*Düsseldorf*: Universitätsklinik (R. Haas, G. Kobbe)
*Erlangen*: Universität Erlangen-Nürnberg (A. Mackensen, B. Spriewald)
*Essen*: Universitätsklinikum (U. Dührsen, A. Hüttmann)
*Essen-Werden*: Kliniken Essen Süd (P. Reimer, F.-K. Baur, M. Wattad)
*Frankfurt*: Universitätsklinikum (H. Serve, N. Gökbuget)
*Frankfurt*: Städt. Klinikum Frankfurt-Höchst (G. Derigs, M. Oestreicher)
*Freiburg*: Universitätsklinikum (M. Lübbert, F. Kaul)
*Gießen*: Universitätsklinikum (M. Rummel, W. Blau)
*Greifswald*: Ernst-Moritz-Arndt-Universität (G. Dölken, C. Busemann)
*Hagen*: Katholisches Krankenhaus (W. Lindemann, V. Rethwisch)
*Halle/Saale*: Martin-Luther-Univ. Halle-Wittenberg (H.-J. Schmoll, H.-H. Wolf)
*Hamburg*: Asklepios Klinik St. Georg (N. Schmitz, R. Stuhlmann)
*Hamburg*: Asklepios-Klinik Altona (D. Braumann, H. Salwender)
*Hameln*: Kreiskrankenhaus (H. Schmidt, F.Krebbel)
*Hamm*: Evangelisches Krankenhaus (J. Schubert, E. Lange)
*Hanau*: Städt. Klinikum (M. Burk, A. Sendler)
*Hannover*: Medizinische Hochschule (A. Ganser, H. Diedrich)
*Heidelberg*: Universitätsklinikum (A. Ho, J. Dengler)
*Homburg/Saar*: Med. Universitätsklinik und Poliklinik (M. Pfreundschuh, G. Held)
*Karlsruhe*: Städt. Klinikum (M. Bentz, S. Wilhelm)
*Kassel*: Klinikum (M. Wolf, B. Ritter)
*Kiel*: Universitätsklinikum, Campus Kiel (M. Kneba, S. Irmer)
*Köln*: Universitätsklinik (M. Hallek, K.-A. Kreuzer)
*Krefeld*: Klinikum (T. Frieling. M. Planker)
*Lemgo*: Klinikum Lippe-Lemgo (F. Hartmann, T. Hesse)
*Leverkusen*: Klinikum (N. Niederle, M. Kreß)
*Lübeck*: Universitätsklinikum, Campus Lübeck (T. Wagner, S. Peters)
*Magdeburg*: Otto-von-Guericke-Universität (T. Fischer, M. Mohren)
*Mainz*: Universitätskliniken (C. Huber, J. Beck)
*Mannheim*: Klinikum (W.-K. Hofmann, N. Härtel)
*Marburg*: Klinikum Lahnberge (A. Neubauer, N. Hagner)
*Minden*: Klinikum (M. Griesshammer, A. Renzelmann)
*München*: Krankenhaus München-Schwabing (C. Nerl, Th. Lipp, S. Zewen)
*München*: Klinikum Rechts der Isar (C. Peschel, F. Schneller)
*Münster*: Universitätsklinikum (W. Berdel, M. Stelljes)
*Nürnberg*: Klinikum Nord (M. Wilhelm, K. Wendelin)
*Offenburg*: Klinikum (F. Hirsch, I. Dresel)
*Oldenburg*: Klinikum (C.-H. Köhne, M. Lange)
*Passau*: Klinikum (T. Südhoff, T. Nitsch)
*Potsdam*: Klinikum Ernst von Bergmann (G. Maschmeyer, A. Scholz)
*Regensburg*: Universitätsklinikum (A. Reichle, D. Ditz)
*Rostock*: Medizinische Fakultät der Universität (M. Freund, C. Junghanss)

*Stuttgart*: Robert Bosch-Krankenhaus (W. Aulitzky, L. Leimer)
*Stuttgart*: Diakonissenkrankenhaus (E. Heidemann, J. Kaesberger)
*Trier*: Krankenhaus d. Barmherzigen Brüder (C. B. Kölbel, G. Pollmeier)
*Tübingen*: Universitätsklinikum (L. Kanz, M. Schmalzing)
*Ulm*: Medizinische Universitätsklinik (H. Döhner, M. Schmid)
*Wuppertal*: Helios-Klinikum (A. Raghavachar, T. Binder)
*Würzburg*: Universitätsklinikum (H. Einsele, M. Topp)
*Zwickau*: Heinrich-Braun-Krankenhaus (U. Kreibich, W. Zschille)

## Supplemental Methods

Statistical analysis

The nonparametric Mann-Whitney U test was applied for continuous variables. The Chi Square test, resp. Fisher test was used for frequency tables. Complete remission (CR) after induction therapy was defined according to standard criteria[2]. Patients who died during the induction period were classified as early death and the remaining patients were classified as failure. Relapse was defined by presence of PB blasts, more than 5% leukemic blasts in BM samples or extramedullary leukemia in patients with previously documented CR. OS was assessed from first day of treatment until death or last follow-up. Remission duration was measured from the date of first CR until the date of relapse. Relapse-free patients were censored at the end of the follow-up, while patients with stem cell transplantation in first CR, patients that left the study or died in CR were censored at the respective dates. Estimated probability of OS and remission duration was calculated using the Kaplan-Meier method, and the log-rank test evaluated differences between survival distributions. The statistical analysis was carried out using the SAS program (SAS-PC, Version 9, SAS Institute, Cary, NC), GraphPad Prism software version 5.0 (GraphPad Software, La Jolla, CA, USA), and IBM SPSS Statistics software version 18 (IBM, Armonk, NY, USA).

## Reference List

1. De Keersmaecker K, Real PJ, Gatta GD, Palomero T, Sulis ML, Tosello V et al., The TLX1 oncogene drives aneuploidy in T cell transformation. Nat.Med. 2010;16(11):1321-7

2. European Leukemia Net., European Leukemia Trial Registry Trial: ALL GMALL 07/2003. 2013; [Accessed September 3, 2013]. http://www.leukemia-net.org/trial/download/public/ALL_GMALL07-03_ShortProtEN.pdf?id=489
